# Picosecond Stabilization of Transferred Charge Carriers at Plasmonic Metal–Molecule Interfaces

**DOI:** 10.1002/anie.202517934

**Published:** 2025-11-02

**Authors:** Daniel Sandner, Katrin Schulz, Andrei Stefancu, John Costello, Reinhard Kienberger, Emiliano Cortes, Hristo Iglev

**Affiliations:** ^1^ Chair for Laser‐ and X‐ray physics E11 TUM School of Natural Sciences Technical University of Munich James‐Franck Str. 1 85748 Garching Germany; ^2^ Nanoinstitut München Fakultät für Physik Ludwig‐Maximilians‐Universität München München Germany; ^3^ School of Physical Sciences Dublin City University Dublin 9 Ireland

**Keywords:** Charge transfer, Energy transfer, Plasmonic nanoparticles, Polaron, Time‐resolved spectroscopy

## Abstract

Plasmonic nanoparticles efficiently absorb light across a broad spectral range, enabling energy transfer to adjacent molecules or semiconductors for photocatalytic applications. However, the nature and timescale of charge carrier involvement in these transfer processes remain a subject of ongoing debate. In this study, we employ broad‐band femtosecond time‐resolved infrared spectroscopy (1100–3000 cm^−1^) as a sensitive probe of free charge carriers to investigate charge transfer dynamics in selected molecules adsorbed on silver nanoparticles. Charge transfer is observed exclusively under resonant excitation of the plasmon and in the presence of adsorbed molecules. Notably, the dynamics of the resulting infrared absorption vary significantly with probe frequency and molecular identity. By applying both Drude and Polaron models, we present compelling evidence that the transferred charge carriers undergo stabilization through solvation and polaron formation. As a consequence, the molecule‐specific time constants for charge back‐transfer extend well beyond the commonly assumed sub‐picosecond regime, indicating a more complex relaxation landscape. Furthermore, the temporal evolution of light‐induced changes in molecular IR modes closely parallels that of the free carrier signal, reinforcing the presence of strong charge carrier–adsorbate interactions.

## Graphical abstract

Charge and heat transfer processes between plasmonic nanoparticles and adsorbed moleculal monolayers are observed using ultrafast infrared spectrocopy. Analysis of free carrier absorption and vibrational signals indicates that overflowing charge carriers are stabilized, extending their lifetime to several picoseconds. Both the charge and heat transfer properties vary significantly depending on the type of adsorbed molecules.

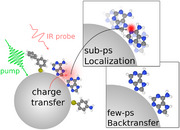

## Introduction

Hot carriers play a key role in photocatalysis, facilitating chemical reactions at ambient conditions, i.e., low temperatures and pressure, improving the reaction selectivity, and ultimately unlocking novel chemical reactions by activating otherwise stable molecular bonds.^[^ ^1^, ^2^, ^3^, ^4^ ^]^ Plasmonic nanoparticles (NPs) combine the advantages of high surface‐to‐volume ratio, minimizing the amount of catalyst required, with the ability to resonantly capture visible light via absorption cross‐sections much bigger than their physical dimensions. These properties make plasmonic NPs highly attractive materials for photocatalysis using solar light.

Following light absorption by excitation of localized surface plasmon resonance (LSPR) modes, the energy is distributed via several steps, on different time and length scales. While early work focused mainly on hot electrons, recent work studied energy partitioning between electrons and holes^[^ ^5^ ^]^ and developed hole‐specific ultrafast methods.^[^ ^6^ ^]^ The plasmon energy can decay through radiative scattering, by scattering photons with the same frequency as the plasmon frequency, or non‐radiatively, by generation of energetic electron‐hole pairs (hot carriers). Once formed, the hot carriers undergo electron–electron scattering and electron–phonon scattering, eventually heating the metal lattice.^[^ ^7^ ^]^ The partitioning between radiative and non‐radiative routes is determined by the dielectric constant of the material and its size and shape. For most catalytic processes, non‐radiative plasmon decay is the relevant decay pathway.

There are three main mechanisms through which LSPRs can catalyze a chemical reactiion. i) Direct, one‐step energy transfer from a LSPR to available resonance states of adsorbed molecules, which is called chemical interface damping.^[^ ^8^, ^9^ ^]^ This corresponds to the fastest energy transfer pathway (10–50 fs), preceding plasmon damping through hot electron generation.^[^ ^10^ ^]^ (ii) Three‐step charge transfer to adsorbed molecules. In the first step hot electrons, e.g., formed by Landau damping,^[^ ^11^, ^12^ ^]^ are transferred to the adsorbate, forming a transient negative ion (TNI), followed by an ultrafast reorganization of the anion's nuclear coordinates, such as bond lengths or angles. Finally, the electron is transferred back to the metal and the adsorbate decays to the electronic ground state, albeit ending up in a vibrationally excited state. The excited vibrational state depends strongly on the intramolecular reorganization and, thus, on the potential energy landscape, making it highly bond‐specific. A single event of scattering and backscattering of a charge carrier into the lowest unoccupied molecular orbital (LUMO) is expected to take place in less than 10 fs. Molecular dissociation or desorption might occur after several such events within the vibrational relaxation time. iii) Hot electrons resulting from plasmon damping couple to phonons through electron–phonon scattering, heating the metal lattice. The associated heat can accelerate chemical reactions due to the exponential dependence with temperature of reaction rate constants via the Arrhenius law, although this process is non‐specific. The temperature gradient is limited by heat diffusion to the substrate and surrounding media, but the enhanced temperature can still increase reaction rates. Temperature effects are expected in the tens of picoseconds to nanoseconds regime in time‐resolved experiments out of equilibrium.

Recently, evidence has accumulated for the second process above. Shin et al. analyzed the overtones of anti‐Stokes peaks in surface‐enhanced Raman spectroscopy (SERS), which showed selective and non‐thermal excitation (up to 500 meV) of molecular bonds, which are, according to calculations, most distorted by the TNI state.^[^ ^13^ ^]^ Christopher et al. have analyzed the influence of different isotopes on the reaction rate and concluded that the smaller mass of ^16^O compared to ^18^O enabled larger distortions within the ultrashort TNI state, causing stronger vibrational pumping and higher reaction rates.^[^ ^14^ ^]^ Stefancu et al. found that engineering of the alignment between the Fermi level and LUMO energies allowed tuning of the vibrational excitation by controlling the scattering of charge carriers between the metal and adsorbed molecules.^[^ ^15^, ^16^ ^]^ However, there is no overall consensus about the nature of energy flow toward adsorbate molecules, and the importance of short‐lived hot carriers versus phonon–phonon scattering (thermal) is still discussed.^[^ ^17^ ^]^ We address this lacuna here by showing that a small spectral range of a few hundred wavenumbers, cannot accurately determine the rate of charge‐carrier back transfer.

Specifically, we use broad band time‐resolved infrared (tr‐IR) spectroscopy to monitor the dynamics of charge transfer, transient ion formation, and the temperature increase of adsorbed molecules on plasmonic Ag NPs. Transferred charge carriers are observed via their broad, Drude‐like, free carrier absorption (FCA). We find that a significant portion of transferred charge carriers resides on adsorbed molecules for several picoseconds (ps). Our measurements indicate that a small spectral range of a few hundred wavenumbers, employed in previous studies, cannot accurately determine the rate of back transfer because a time‐dependent Drude collision time causes the absorption spectrum of the charge carriers to change, requiring tr‐IR spectroscopy in a broad range. Our finding of long‐lived, transferred charge carriers on the adsorbed molecules is corroborated by shifts in vibrational modes that appear within the instrument response function (IRF) and decay within the time of back transfer. The temperature of the adsorbed molecules is monitored via changes in the infrared spectrum and is found to increase within a few to tens of picoseconds, featuring a strong variation across different species of adsorbed molecules.

This article is structured as follows: we first introduce the experimental setup, then discuss the broad induced absorption associated with free carriers, before analyzing transient absorption (TA) in the vicinity of IR modes, distinguishing between thermal and non‐thermal signals.

## Results and Discussion

**Figure 1:**
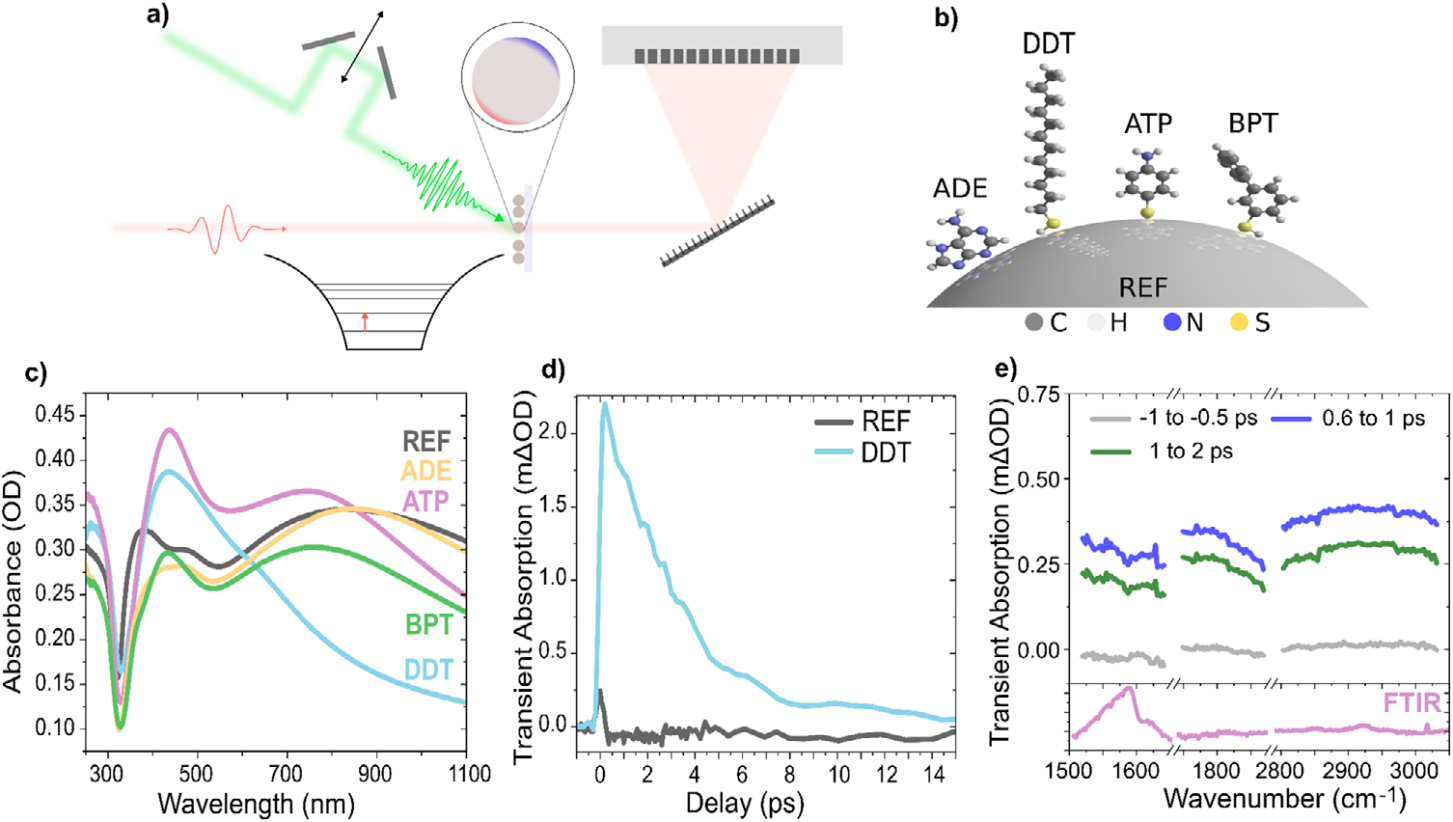
a), Sketch of the pump‐probe experiment. b), Structure of molecules adsorbed to Ag NPs. c), UV–vis extinction spectra of the samples, featuring a LSPR centered at 470 nm. d), Kinetics of the photoinduced absorption for a sample with adsorbed DDT molecules and pure NPs (REF) at a probe frequency of 1400 cm^−1^. Positive TA means increased absorption in the excited state throughout this manuscript. e), Transient absorption spectra of ATP‐covered Ag NPs at several spectral positions, showing photoinduced absorption at positive pump‐probe delays, independent of probe frequency. The lower panel shows FTIR (IR absorption) for comparison.

Before discussing the results in detail, we briefly introduce the pump‐probe experiment and the choice of the optical frequencies. The experiment is shown schematically in Figure 1a: A short green laser pulse, spectrally tuned to the LSPR resonance above 500 nm, excites the Ag NPs. Importantly, the adsorbate molecules, unlike commonly used dye molecules, are not excited by the pump beam, as shown by UV–vis absorbance spectra of the pure adsorbates in Section S1. The pump fluence was between 0.5 and 4 mJ cm^−2^, comparable with recently reported time‐resolved studies.^[^ ^18^ ^]^ A time‐delayed mid‐IR beam, generated via non‐linear optics from the same ultrafast laser source as the pump beam, is used to measure the transmission spectrum by a grating‐based spectrometer equipped with a multi‐pixel detector. Pump‐induced changes in the sample are detected by chopping the pump beam to half the repetition rate and comparing probe intensities with excitation I*(*v*) to those without excitation *I*_0_(*v*). The spectrally resolved *TA*(ν) is calculated according to the formula: *TA*(ν)  =   − *log*[I*(*v*) / *I*_0_(*v*)] and the instrument response function is 150 fs. The pump beam was always tuned to the low‐energy side of the LSPR resonance (520–550 nm) to avoid direct excitation of the metal and give better comparability with commonly employed visible light sources in SERS measurements. The choice of the mid‐IR probe frequency is based on the following consideration: visible probe light, close to the plasmon resonance, shows intense changes in transmission as the excitation and subsequent heating of the electrons in Ag NPs causes substantial broadening of the plasmon resonance.^[^ ^18^ ^]^ This photo‐induced change in the dielectric function of the NPs is far more potent than the contributions we expect for the adsorbates, which are not optically active in the visible range. In the mid‐IR range, the opposite is true: Ag NPs in the studied size range show negligible, spectrally unspecific absorption. At the same time, the adsorbed molecules feature characteristic and narrow vibrational transitions. Compared to time‐resolved SERS, the mid‐IR probe has less intensity and doesn't excite the plasmon resonance.^[^ ^19^ ^]^ In addition, similar to THz spectroscopy, mid‐IR light is subject to free carrier absorption and thus sensitive to free charge carriers.^[^ ^20^ ^]^

Figure 1b shows the four different molecules 4‐aminothiophenol (ATP), 1‐dodecanethiol (DDT), 4‐biphenylthiol (BPT), and adenine (ADE) adsorbed onto Ag NPs. We compared the optical response of these four systems with a sample containing only the Ag NPs (REF). The synthesis and preparation of the Ag NPs samples is detailed in Section 1 of the Supporting Information. Importantly, all of these molecules are bound to the metal surface by chemisorption and form ordered, self‐assembled monolayers (SAM).^[^ ^21^, ^22^ ^]^

To tune the pump frequency, we have extracted the absorbance of all samples from transmission measurements (displayed in Figure 1c). Note that reflection and scattering of light also appear as extinction. The main peak at ∼470 nm with a width of 1.4 eV (FWHM) is commonly assigned to the LSPR for NPs of the employed size.^[^ ^23^ ^]^ Additional absorbance peaks between 700 and 1000 nm are most likely a result of NP aggregation during deposition,^[^ ^24^ ^]^ as indicated by the lack of these features in the extinction spectra of the same NPs in solution (see Figure S4).

### Spectrally Broad Mid‐IR TA

Figure 1d shows pump‐probe transients at a frequency of 1400 cm^−1^ for pure Ag NPs (black line) and those functionalized with DDT (cyan line). The latter shows positive TA, meaning increased absorption after excitation, which is assigned to FCA of transferred charge carriers.^[^ ^2^, ^20^ ^]^ The transient signal in pure Ag NPs is weaker and shows the opposite sign than that of DDT at longer times. The peak at zero delay time has the same width as the cross‐correlation of pump and probe pulses and is attributed to non‐linear χ^(3)^ interactions (sometimes referred to as a coherent artifact).^[^ ^25^ ^]^ Without adsorbates, we don't expect charge transfer and assign the small, increased transmission (i.e., negative TA) to a reduced scattering of the probe light. The reflectivity of metals is known to decrease at high (electron or lattice) temperatures.^[^ ^26^ ^]^ Additional experiments were performed with an excitation wavelength of 800 nm to investigate the nature of broad absorption features in the NIR, presumably a result of NP aggregation. As shown in Figure S16, the pump‐induced IR absorption was very weak and of negative sign. This observation agrees with previous studies that observed the strongest hot‐carrier‐mediated catalysis and charge transfer for resonant LSPR excitation.^[^ ^27^, ^28^, ^29^ ^]^ TA has been measured for probe frequencies between 1100–3000 cm^−1^. Since the probe beam has insufficient spectral width to cover this range at once, the central probe frequency was shifted several times, and TA was recorded with a high spectral resolution of a few wavenumbers in a narrow range. Figure 1e shows three pump‐probe measurements of Ag NPs covered with ATP. Negative pump‐probe delays (black line) act as a baseline as the probe beam passes the sample before the pump beam, prohibiting pump‐induced changes in absorption. Following excitation, at positive delays, we observe an increase in absorption. The broad and unspecific spectrum of this absorption rules out a vibrational origin and suggests the presence of free charges.

**Figure 2:**
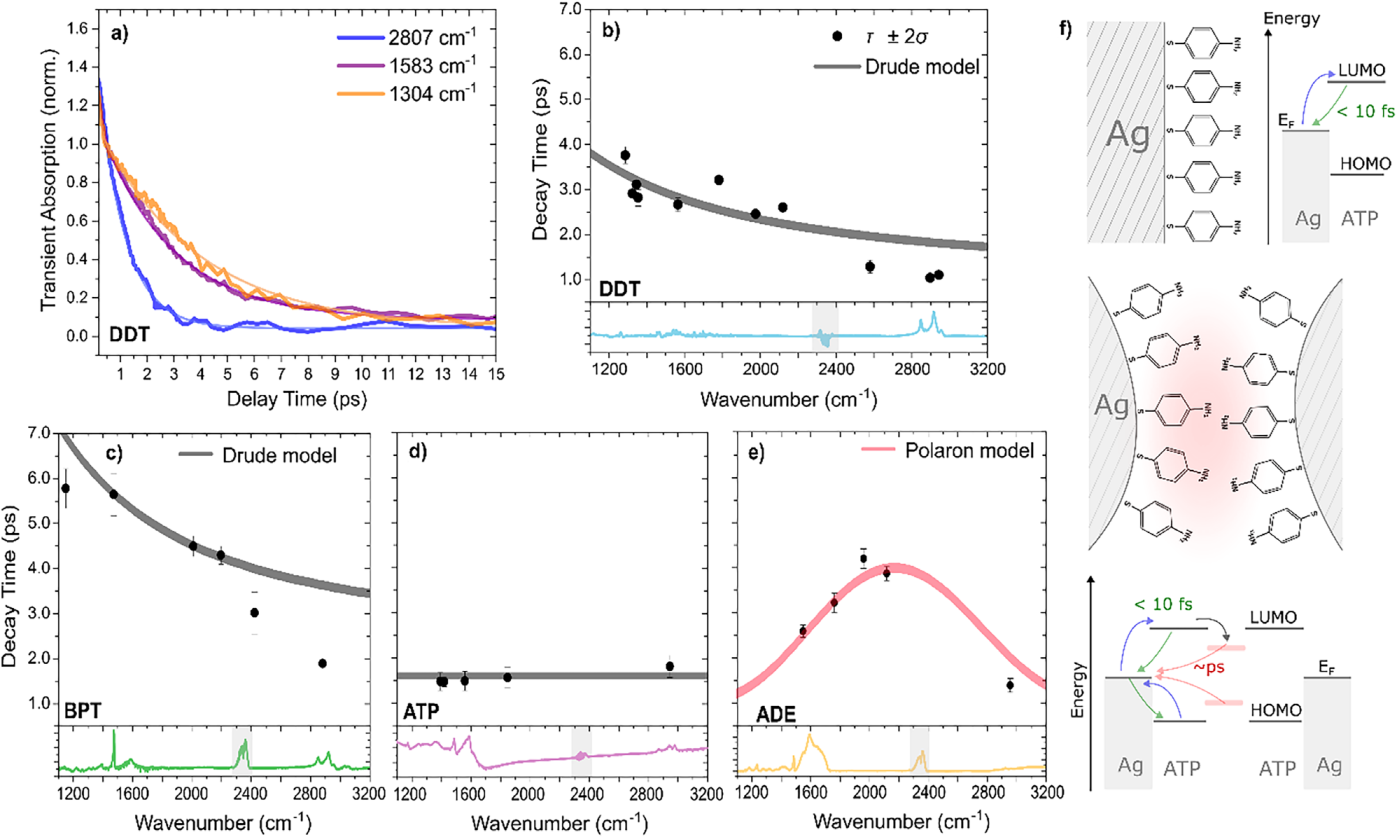
a), Transients of DDT‐covered Ag NPs for different probe frequencies with mono‐exponential fits. b)–e), Extracted decay constants versus probe frequencies for all studied samples. Lower panels show IR absorption; grey bands indicate  atmospheric CO_2_ absorption. f), Sketch of trap states/solvation in the adsorbate layer allowing picosecond lived transferred charge carriers.

TA of NPs with adsorbates was fitted with a single exponential decay convoluted with the IRF. Since time constants extracted by exponential fits are only meaningful for monomolecular decays, we performed pump‐fluence‐dependent measurements. We obtained the same decay constants in the studied fluence range from 0.5 to 4 mJ cm^−2^ (see Section 3 in the Supporting Information), justifying the monomolecular exponential fit. Stretched exponential fits were also tested and showed a stretching factor close to one (see Section 3.4 in the Supporting Information), consistent with homogeneity at the relevant length scale of the metal–molecule interface. Importantly, the fluence‐independent dynamics enable us to neglect the effect of inhomogeneous excitation across the varying film thickness. We don't expect a change in dynamics for different probe frequencies in the simplest model of transferred charge carriers with a finite lifetime, absorbing light in the THz and IR range. However, the decay dynamics, shown in Figure 2, exhibit substantial variations depending on the sample and probe frequency, as demonstrated for DDT by sample curves and the extracted decay constants in Figure 2a,b, respectively. BPT and DDT show shorter decay constants at higher probe frequencies, while ATP shows no significant probe‐frequency dependence, and ADE shows a peak‐like distribution of time constants centered at 2000 cm^−1^. Pump‐probe transients are shown for all samples in Section 3 in the Supporting Information. We want to emphasize that the vibrational modes don't affect the decay constants shown in Figure 2. Because of the narrow spectral width of molecular vibrations, excited state signals are easily identified. Most vibrational modes didn't show photo‐induced changes, and the changes in absorption are an order of magnitude weaker than the broad background, whose dynamic is shown in Figure 2.

The response of free charges is commonly analyzed in mid‐IR and THz spectroscopy by fitting the TA spectrum with the (generalized) Drude model.^[^ ^30^, ^31^ ^]^ This approach cannot be applied directly to our data analysis, as the TA spectrum between 1100 and 3000 cm^−1^ was recorded not simultaneously but rather through multiple measurements. Comparing the TA amplitudes across different measurements is challenging because the spatial overlap of pump and probe beams can shift during alignment adjustments used to change the probe frequency. Additionally, the probed region on the sample varied between measurements, leading to local fluctuations in the NP density that linearly influence signal strength and cause variation up to 300% in TA amplitudes (see Figure S7). Instead, the probe‐frequency dependent decay constant has proved to be a more reliable parameter, as it remains unaffected by sample position and the pump fluence, which also fluctuates with changes in the spatial pump‐probe overlap (see Section 3.1 of the Supporting Information for dynamics and amplitudes obtained at different sample positions).

Therefore, we analyze our data using the Drude model, however, rather than relying on the absolute absorbance of the charge carriers, we focus on the model's predicted time dependence as a function of probe frequency. To develop an intuitive understanding of this approach, one can assume the initial TA spectrum at 100 fs is relatively flat. Consequently, the distribution of decay constants should resemble the TA spectrum (in amplitude) observed after a few picoseconds.

The complex dielectric function in the Drude model is:

(1) $$\varepsilon \left(\omega \right)=\varepsilon_{\infty }-\frac{\omega_{p}^{2}}{\omega^{2}+i\omega /\tau_{D}}$$

with plasma frequency $\omega_{p}^{2}=\frac{Ne^{2}}{\varepsilon_{0}m_{eff}},$ charge carrier density *N*, effective mass *m_eff_*, and Drude collision time τ_*D*_. The high‐frequency dielectric constant ε_∞_ represents the non‐vanishing flat contributions of other transitions (e.g., phonons or transitions in the visible). For light absorption, we need to consider the absorption coefficient α(ω):

(2) $$\alpha \left(\omega \right)=\frac{\omega }{\mathrm{nc}}\varepsilon^{\prime \prime }\left(\omega \right)=\frac{\omega_{p}^{2}}{\mathrm{nc}\;\tau_{D}\left(\omega^{2}+1/\tau_{D}^{2}\right)}$$

where ε′′(ω) represents the imaginary part of ε(ω), *n* is the refractiv index, and *c* is the speed of light. From the equations above, we can see that the time‐dependent dynamics of *N(t)*, including excitation and recombination of charge carriers and the time‐dependence of *m_eff_*, don't contribute to the probe‐frequency dependent dynamics. The frequency dependence of decay constants is only obtained by accounting for the time‐dependence of the Drude collision time τ_*D*_, affecting the spectrum of Drude absorption.

Thus, TA data for ATP‐covered NPs, shown in Figure 2d, can be explained by the simplest model of electron back transfer within (1.5 ± 0.1) ps, τ_*D*_ being constant. In contrast, for DDT‐covered Ag‐NPs shown in Figure 2b, we extract a change in τ_*D*_ from 2 fs to (19 ± 6) fs within 1.1 ps and a recombination/back transfer time of 5.6 ps (see Section 3.5 in the Supporting Information for a detailed description of modeling and fitting). The same evolution in τ_*D*_ has been previously reported for optically excited semiconductors.^[^ ^32^ ^]^

The limitations of the Drude model are most pronounced in spatially confined systems, such as charge carriers interacting strongly with the vibrational modes. Equation 1 shows that the Drude model predicts monotonically decreasing absorbance with increasing frequency. Further, time dependence in τ_*D*_ causes monotonical trends in the observed decay constants. Therefore, the Drude model cannot explain the dynamics of ADE‐covered NPs, exhibiting the slowest decay at ∼2000 cm^−1^. Two groups of models are commonly employed to describe systems with semi‐free charge carriers. Smith has modified the Drude model with a parameter describing localization, causing a drop in conductivity (and absorption) toward ω = 0 because of the back‐scattering of carriers.^[^ ^33^ ^]^ The Drude–Smith model has been refined further and is commonly applied to describe the THz response of nanoscale semiconductors and molecular networks.^[^ ^34^ ^]^ Another description is given by polaron theory, which introduces polarons as quasi‐particles resulting from the interaction of a charge carrier with molecular/phononic vibrations and characterizes the coupling via a binding energy, *E_pol_*.^[^ ^35^ ^]^ Independent of the chosen model, we can qualitatively state that charge carriers transferred to ADE molecules exhibit stronger spatial confinement than all other molecules studied herein. In its simplest form, the Polaron model requires fewer parameters, and we can quantitatively extract a transition from a Drude‐like spectrum to a Polaron‐like response within one ps and a decay constant of 6.8 ps for recombination. In the small polaron picture, we find a binding energy of (0.10 ± 0.02) eV. Because of the lack of TA data below 1100 cm^−1^, a limit imposed by the CaF_2_ substrate, we cannot distinguish between the Drude‐like and Polaron‐like models for BPT‐covered NPs.

We emphasize the pronounced adsorbate‐specific time dependence of the spectral response of transferred charge carriers. The presented model is a simple extension to the well‐known Drude model by adding another time‐constant. We expect that future studies can extend this description by observing and analyzing the amplitude distribution of the broad FCA and modeling it in the context of hybridized metal–molecule states. In summary, charge–carrier–adsorbate interactions depend strongly on the given molecule, allowing for tuning of the timescale and energy landscape of the excited state following plasmon damping.

For plasmonic NPs coupled to semiconductors, charge transfer and back transfer on the pico‐to‐nanosecond timescale have been reported several times. Long lifetimes can be explained by a Schottky barrier between metal and semiconductor,^[^ ^28^, ^36^ ^]^ and high yields have been ascribed to direct excitation of the conduction band through plasmon damping.^[^ ^37^ ^]^ However, adsorbed molecules on plasmonic NPs require a different physical description involving single molecular orbitals instead of hybridized energy bands. In addition, one needs to consider the differences between the commonly employed plasmonic metals Ag and gold (Au). Only in the latter can photon energies in the visible range excite interband excitons with a hole in the d‐band and electrons in the sp‐band.^[^ ^38^ ^]^ Similarly, plasmons can decay by exciting the interband electron–hole (e–h) pair, which has a longer lifetime of ∼1 ps compared to intraband excitons, which recombine in ∼100 fs.^[^ ^39^ ^]^ For Au NPs, chemical reactions involving more than one electron have been reported in the presence of hole scavengers, indicating long‐lived electrons on the adsorbed molecules.^[^ ^39^, ^40^ ^]^ In the time‐domain, Contreras et al. have observed an ultrafast (∼50 fs) charge transfer from the Fermi level of Au NPs to the LUMO of methylene blue with a lifetime of ∼1 ps.^[^ ^41^ ^]^ To our knowledge, there is little evidence on ps‐lived charge carriers in molecules adsorbed to Ag NPs. It is often assumed, that charge transfer in Ag NPs consists of the ultrafast formation of a TNI with a lifetime of few fs^[^ ^42^ ^]^ and vibrational excitation following the backscattering from the LUMO to the metal (DIET/DIMET‐ desorption induced by (multiple) electron transitions). We note that for polar small molecules, mainly water but also ammonia or DMSO, picosecond‐lived charge carriers and electron solvation have been observed on metal surfaces.^[^ ^43^, ^44^, ^45^, ^46^ ^]^

From our experimental observations, we cannot provide an unambiguous explanation for the long lifetime of charge carriers on the adsorbate molecules. We conjecture that localization or polaron formation, meaning a structural reorganization of the adsorbate layer creating lowered electronic states, plays an important role, but other effects could give similar results. Notably, most experiments on the ultrashort lifetime of TNIs were performed on well‐defined, monolayer‐covered metal surfaces in a high vacuum (top panel of Figure 2f).^[^ ^42^ ^]^ For the deposited NPs studied here, one can assume local minima in ensembles of coated NPs, e.g. between adsorbate layers of different NPs. Electrons or holes scattered in these regions would cool rapidly and lose overlap with states close to the Fermi edge, with both effects increasing the Drude collision time. The minimum being local would lead to a stochastic decay of the trapped population via back‐transfer to the NP (see middle and lower panel in Figure 2f). A similar mechanism could also be expected for diluted, adsorbate‐covered NPs in solution due to the adsorbate–solvent interaction and electron solvation, as well known for water.^[^ ^47^, ^48^, ^49^, ^50^ ^]^ We want to emphasize that the concept of solvation is most studied for electrons as in water, but it works similarly for positively charged particles or holes.

Most experiments on plasmonic NPs are performed under continuous excitation, where the lifetime of transferred charge carriers is not directly observable but may influence the studied properties, like vibrational excitation or chemical reaction rate. We expect that the long‐lived transient ions found here show negligible isotope effects as the picosecond lifetime allows molecular bonds to change in length and angle to the full range determined by the potential energy landscape. In contrast to the commonly discussed short‐lived TNI states, the distortion and corresponding vibrational excitation should not depend on the initial acceleration determined by the atomic mass. An immediate consequence of our work is that future studies aiming to follow transferred charge carriers via infrared light^[^ ^2^ ^]^ could employ multiple probe frequencies to assess the time of back‐transfer.

### Vibrational Response

Having discussed the spectrally broad absorption changes after excitation, caused by free charge carriers, we now focus on spectrally narrow vibrational signals (IR modes) in the TA spectra. We have only observed distinct TA signals in the vicinity of IR modes of the unpumped sample studied via FTIR. Many effects can influence the oscillator strength, resonant frequency, and lineshape of infrared absorption modes. Therefore, the assignment of photo‐induced changes is often ambiguous. Molecular vibrations are only IR active if the change in nuclear coordinate by the vibration is associated with a change in the electric dipole moment. Hence, changes in the charge density, e.g., by nearby charge carriers, can reduce the strength of an IR mode (infrared amplitude modulation, IRAM) or enhance previously IR‐inactive vibrations (infrared activated vibration IRAV).^[^ ^51^ ^]^ In addition, conformational changes in bond lengths and angles, as required by the DIET mechanism, can change the strength and resonant frequency of the vibrations. Structural reorientation in molecules can occur as fast as the vibration period, meaning all IR modes investigated here can respond faster than ∼30 fs, which is a fraction of IRF (∼150 fs). Likewise, vibrational excitation typically causes redshifts by anharmonicity. Small shifts in peak position and lineshape due to changes in the dielectric environment are also described by the vibrational Stark effect^[^ ^52^ ^]^ and via Fano theory.^[^ ^53^, ^54^ ^]^

**Figure 3:**
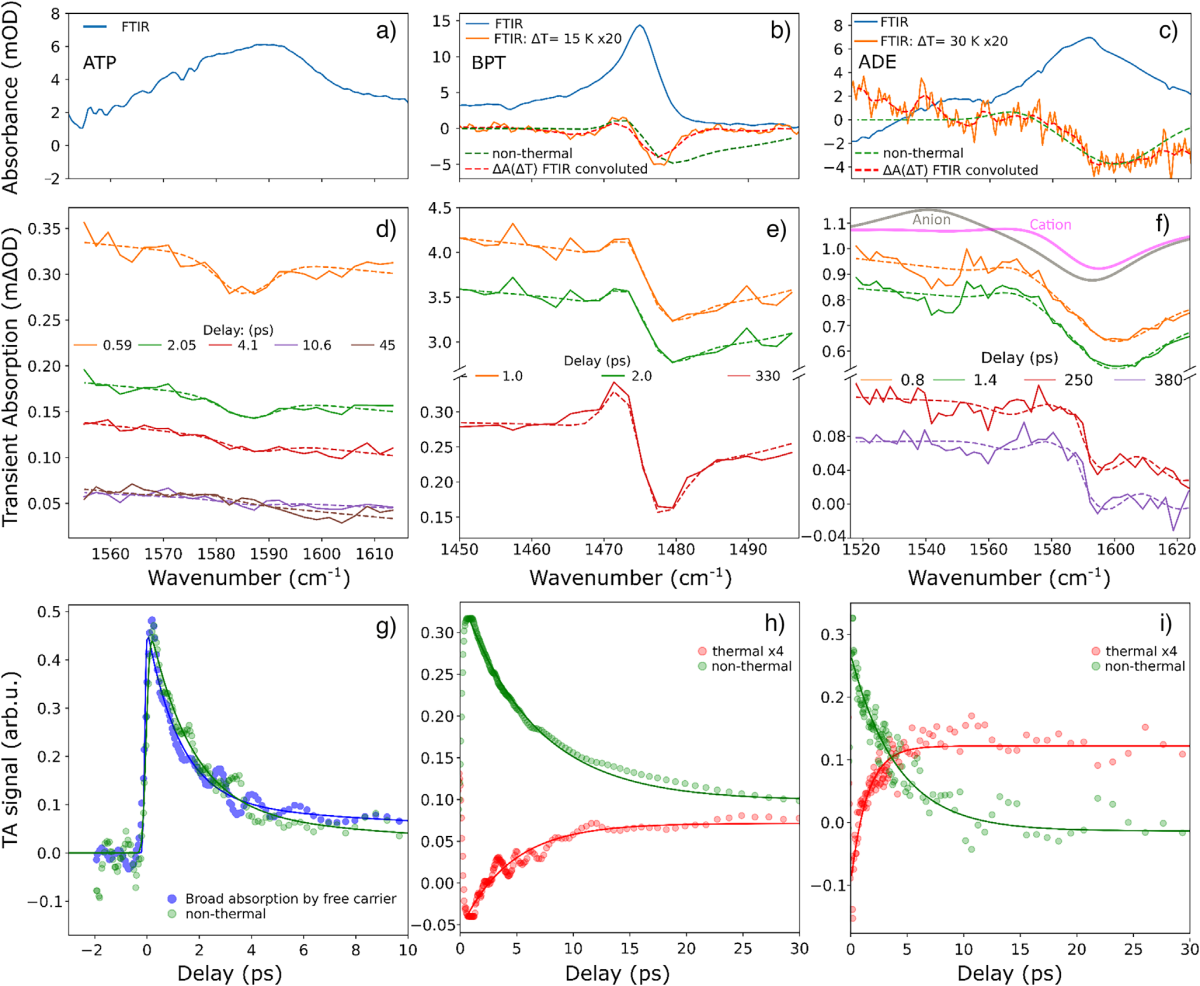
FTIR spectra and temperature‐induced difference for ATP, BPT, and ADE in a)–c). TA spectra at various delays for the same molecules in d)–f) together with calculated difference spectra in (f). The TA spectra (solid) are fitted (dashed) by the sum of a non‐thermal lineshape (approximated below 1 ps) and a thermal lineshape (by temperature‐dependent FTIR). g)–i), Dynamics of the amplitudes associated with the respective lineshape as determined by fitting of the TA spectra (dots) with exponential fits (solid lines).

Figure 3a shows an infrared mode of ATP‐covered NPs at ∼1590 cm^−1^ assigned to C─C stretching in the aromatic ring.^[^ ^55^ ^]^ The inhomogeneously broadened lineshape suggests a combination of bands. In the TA spectra shown in Figure 3d, we observe bleaching at 1585 cm^−1^, which is strongest at small delays and doesn't match the very weak temperature‐induced difference spectrum (see temperature‐dependent FTIR in Section S4). We extracted the magnitude of the narrow bleaching via curve fitting of TA spectra with a peak function of fixed width, including a straight background for broad absorption. Figure 3g shows the time‐dependent amplitude of the bleaching as extracted by fitting (green dots) and the broad absorption in the same probe‐frequency range (blue dots). Notably, the vibrational response decays with the same time constant of (1.7 ± 0.2) ps as the transferred charge carrier population (c.f. Figure 2d). This indicates that the IR mode at 1590 cm^−1^ is a local reporter for the transferred charge carriers. A comparison between the TA spectra and calculated IR spectra of ions indicates electron transfer from the ATP layer toward the Ag NP, as expected from the energy alignment of HOMO and LUMO levels (see Sections S5 and S6).

Figure 3b shows the IR peak at 1475 cm^−1^ of BPT‐functionalized Ag NPs. The peak can be attributed to the aromatic C─C stretching mode^[^ ^55^ ^]^ and shows a weak redshift upon heating (orange curve). The analysis of TA spectra is more complicated than for ATP because the photoinduced transient signal (see Figure 3e) has a lineshape similar to the temperature‐induced absorbance change and shows a long‐lived component that doesn't decay in the studied range (up to 500 ps). This motivates the following analysis: Absorbance change due to a temperature jump Δ*A*(Δ*T*) is extracted from temperature‐dependent FTIR measurements (orange curve in Figure 3b) and convoluted with the spectral resolution of the grating spectrometer used for the time‐resolved experiment, yielding the red dashed line in Figure 3b. For the carrier‐mediated response, we modeled the TA spectrum below 1 ps using multiple Gaussian peaks (see green dashed line in Figure 3b). All acquired TA spectra were fitted as a superposition of these two spectral shapes with individual amplitudes, which are shown in Figure 3h. As expected, the amplitude extracted for the thermal lineshape increases over several ps. It reaches a plateau, which is maintained until 500 ps due to slow heat diffusion. The rise time, determined by a mono‐exponential fit, is (4.4 ± 0.3) ps, in good agreement with recent studies.^[^ ^56^ ^]^ The amplitude extracted for the non‐thermal lineshape decays exponentially with a time constant of (6.5 ± 0.1) ps, which matches the longest‐lived broad electronic absorption, as shown in Figure 2c, within the errors. Transients were fitted starting at 0.2 ps to avoid artifacts by perturbed free induction decay (PFID). Interestingly, the non‐thermal TA spectrum is characterized by bleaching in a region with little IR absorption of the ground state. This suggests that instead of the bleaching of an IR mode on top of the broad absorption, this signal is a modulation of the broad absorption due to a Fano anti‐resonance (two excitation pathways at the same frequency interfering destructively). The same mechanism has been recently described in detail for charge transfer in similar systems.^[^ ^53^ ^]^

Figure 3c shows the infrared absorption and its temperature dependence of an in‐plane N─H bending mode of ADE‐covered Ag NPs at 1590 cm^−1^.^[^ ^57^ ^]^ As discussed for BPT, we observe a long‐lived vibrational response in the TA spectra (see Figure 3f), which we explain by the temperature increase. Similar to BPT‐covered Ag NPs, we used two lineshapes to describe the TA spectra (see green and red dashed lines in Figure 3c), with the thermal spectrum being the temperature‐induced change found in the FTIR spectrum convoluted with the spectral resolution of the time‐resolved setup. The analysis of the extracted amplitudes reveals an even faster rise in temperature of (1.5 ± 0.1) ps. Note that other bonds in the same molecule may show a slower rise in their thermal response depending on the specificity of electron–phonon and phonon–phonon coupling. The non‐thermal vibrational response decays with a time constant of (4.1 ± 0.2) ps, in good agreement with the longest observed broad IR absorption shown in Figure 2e. Importantly, the free carrier response at 1600 cm^−1^ decays twice as fast, requiring spectrally broad IR absorption to connect vibrational and FCA dynamics. The comparison between the non‐thermal spectrum and model calculations, shown in Figure 3f, is in favor of electron transfer toward the Ag NP (or hole transfer from the NP to the adsorbant), leaving the molecule in a cation state, as expected from the Fermi‐level‐HOMO–LUMO alignment (see Sections S5 and S6).

In summary, we observed vibrational responses in TA spectra, which allowed us to distinguish thermal and non‐thermal contributions by their lineshape and temporal characteristics. For the non‐thermal signals rising within the IRF, we find that the decay times match the longest‐lived broad electronic signals, even if they were recorded at different probe frequencies. This is consistent with the picture of ps‐lived charge carriers residing on the adsorbate molecules. In addition, we found that the dynamics of the adsorbate‐temperature increase are also highly molecule‐specific, ranging from 1.5 ps for ADE to 14 ps in DDT (see Section 4 in the Supporting Information). Microscopically, this rise time is determined by the coupling between the phonon modes of the adsorbate and the silver NP, and the heat capacity of the adsorbate layer. Considering the latter, we calculated single‐molecule thermal conductances of (160 ± 10), (75 ± 5), and (55 ± 5) pW K^−1^ for ADE, BPT, and DDT, respectively. This further highlights the differences at the metal–molecule interfaces between thiolated and non‐thiolated adsorbates. Because the experimental findings of this study are related to the dynamics of carriers transferred to the adsorbate layer on the picosecond timescale, without discussing absolute amplitudes and yields, we assume that we are insensitive to ultrafast processes such as hot carrier generation inside the Ag NPs, which are known to be size and shape dependent.

## Conclusion

We applied time‐resolved infrared spectroscopy to adsorbate‐functionalized Ag NPs and measured an increased absorption after exciting the LSPR, indicating metal–molecule charge transfer. The decay of the transient signal, typically assigned to recombination or back‐transfer, depends strongly on the probe frequency and adsorbate, implying changes in the absorbance spectrum of transferred charge carriers, consistent with extended Drude models or Polaron theory. Depending on the molecules at the Ag surface, we find back‐transfer times of 1.6–6 ps, highlighting the importance of charge carrier–adsorbate and adsorbate–adsorbate interactions. The picosecond lifetime of transferred charge carriers is further observed in the vibrational response of several molecular bonds, showing red‐shifts and bleaching. The distinct temporal behavior and spectral lineshapes enable us to distinguish charge‐mediated change in IR absorption from thermal effects. The analysis of thermal signatures reveals a rise in adsorbate temperature between 1.5 and 14 ps. Therefore, in this study, we highlight the potential of time‐resolved mid‐IR spectroscopy to track energy transfer between plasmons in noble metal (e.g., Ag, Au, Pt) NPs and adsorbed molecules. Understanding this process can ultimately be used to design functionalized NPs for plasmonic catalysis.^[^ ^58^ ^]^ Further studies under reactive conditions may also find new emerging IR modes due to bond formation in chemical reactions. Comparison of the reaction product formation time with the timescales of charge carrier back‐transfer and adsorbate cooling will directly point toward the dominant contribution in plasmonic catalysis. Moreover, we show here that, unlike the common assumption, transferred charge carriers can have relatively long lifetimes–reaching even several ps–presumably by solvation/polaron formation, enhancing plasmon‐driven chemical reactivity.

## Conflict of Interests

The authors declare no conflict of interest.

## Supporting information



Supporting Information

## Data Availability

The data that support the findings of this study are available from the corresponding author upon reasonable request.
