# Supplementary material for: Picosecond Stabilization of Transferred Charge Carriers at Plasmonic Metal–Molecule Interfaces
Source: Angew Chem Int Ed Engl. 2025 Nov 2;65(1):e17934. doi: 10.1002/anie.202517934 (PMC12759206; doi:10.1002/anie.202517934)
Supplement: Supplementary file 1 — Supporting Information [file ANIE-65-e17934-s001.pdf]

Supporting Information for

Picosecond stabilization of transferred charge carriers at plasmonic metal-molecule interfaces

Daniel Sandner[+,1], Katrin Schulz[+,1], Andrei Stefancu[2], John Costello[3], Reinhard Kienberger[1], Emiliano Cortes*[,2] and Hristo Iglev*[,1]

[+]Equal contribution

*Authors to whom the correspondence should be addressed:

Hristo Iglev, hristo.iglev@tum.de

Emiliano Cortes, emiliano.cortes@lmu.de

[1] Chair for Laser- and X-ray physics E11, TUM School of Natural Sciences, Technical University of Munich, James-Franck Str. 1, 85748 Garching, Germany.

[2] Nanoinstitut München, Fakultät für Physik, Ludwig-Maximilians-Universität München, München, Germany.

[3] School of Physical Sciences, Dublin City University, Dublin 9, Ireland.

Contents

Section 1: Sample Synthesis and Characterization 2
Section 2: Time-resolved IR spectroscopy: 4
Section 3: Probe-frequency dependent dynamics 7
Section 4: Thermal changes in IR absorption 17
Section 5: Energy alignment of molecules with silver 20
Section 6: Non-thermal (ultrafast) vibrational spectra: 21
Section 7: Role of adsorbed water 24
References 25

Section 1: Sample Synthesis and Characterization

AgNPs capped with citrate anions, synthesized by Lee and Meisel method[1], were used throughout this study. Briefly, the AgNPs were synthesized as follows: in 98 mL ultrapure water, 17 mg $AgNO_3$ were dissolved. The solution was boiled under constant magnetic stirring. At boiling, 2 mL of a trisodium citrate solution (1%) were added dropwise and the solution was left to boil for another hour.

Next, the AgNPs were deposited on the CaF_2 substrates through convective self-assembly. First, 2 mL of the AgNPs colloidal solution was concentrated by centrifugation at 7300 × g, for 15 min. The supernatant was discarded by pipetting, and the AgNPs were resuspended in 20 µL ultrapure water. The CaF_2 substrates were thoroughly cleaned by ultrasonication in acetone before the deposition of the AgNPs and were placed in a UV-Ozone cleaner for 15 min prior to the deposition of the AgNPs. The UV-Ozone irradiation has a dual role: first, it cleans the substrate from organic impurities, and second, it renders the surface hydrophilic and decreases the surface tension of the colloidal AgNPs drop, so that it spreads more easily. The CSA coater comprised of a motorized translational stage (Thorlabs Inc.) that uses a linear actuator from Thorlabs. A regular microscope slide that acted as a blade was fixed in the near vicinity of the substrate at the desired angle, while the concentrated AgNPs solution was placed on the substrate, underneath, and nearby the edge of the blade. More details about this technique can also be found in refs. [2-3]. For the deposition of the AgNP, the motorized table was programmed to move continuously at a speed of 0.05 mm s^{-1} during which the colloidal AgNPs deposit on the substrate.

The AgNPs substrate was functionalized with the four molecules by immersing it overnight in a 1 mM solution. Afterwards, the substrate was rinsed with ethanol or water (in the case of Adenine).

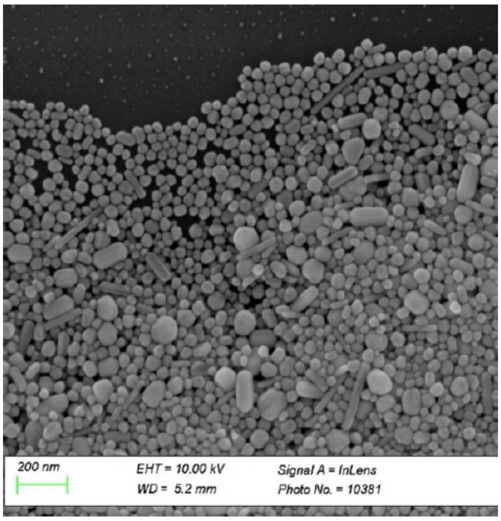

Figure S1. SEM image of the Ag NPs film.

We would like to note that the size of the probe and pump beams at several tens to hundreds of micrometers leads to an averaging over NPs in a broad range of sizes, diminishing highly size-selective effects. The macroscopic homogeneity of our samples is demonstrated below in Figure S7.

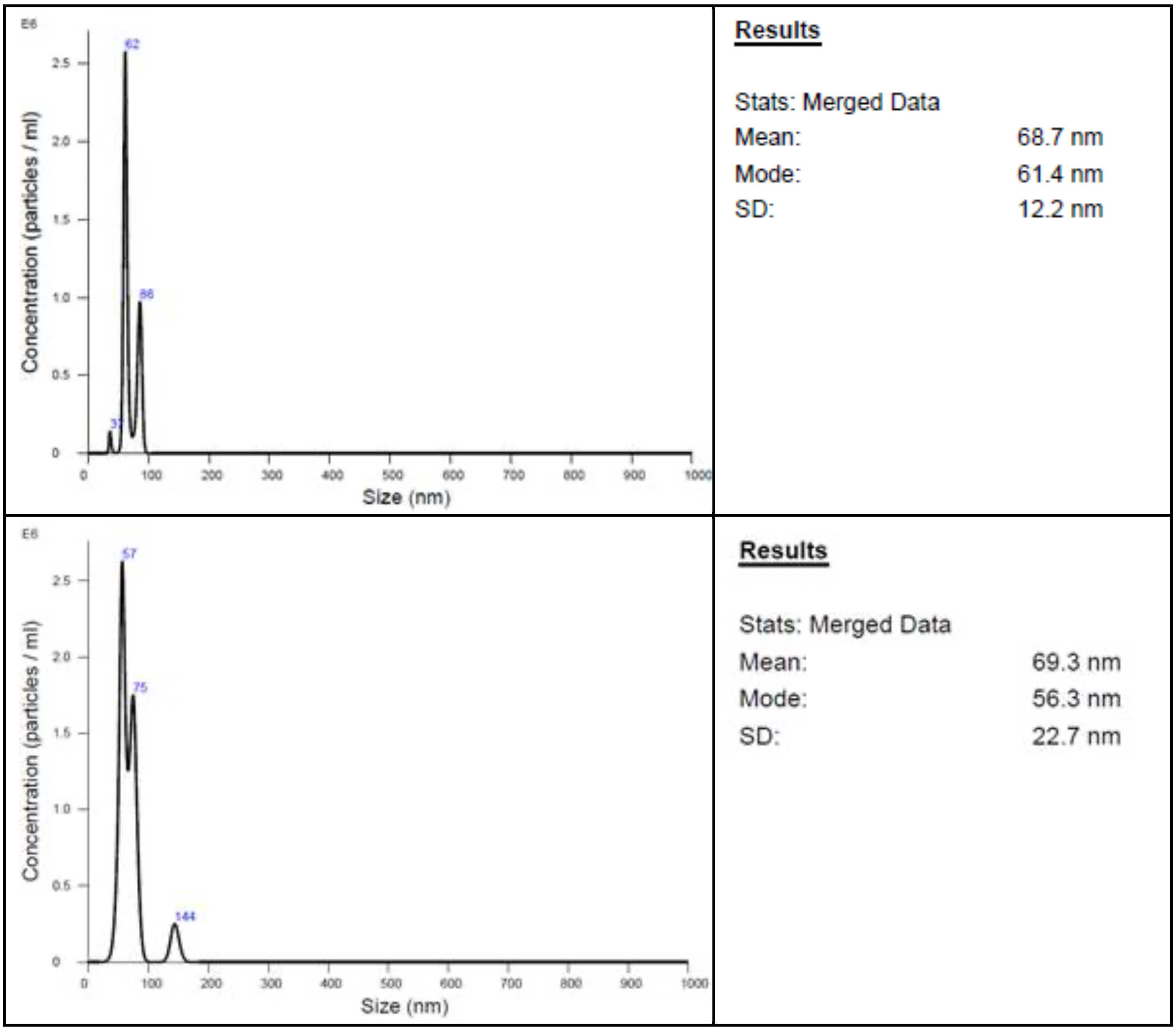

Figure S2: Nanoparticle tracking analysis of the AgNPs for two separate colloidal solutions.

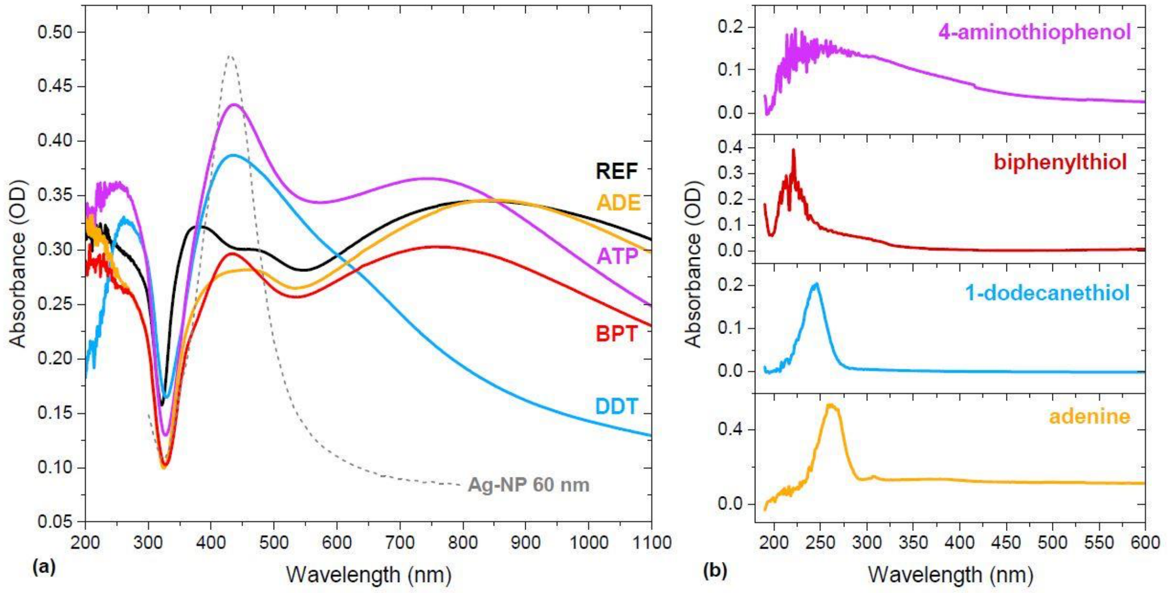

Figure S3 a: UV-Vis absorbance of adsorbate-covered NPs, b: Absorbance of pure adsorbates. Excitation wavelength around 520 nm.

Figure S3b demonstrates that excitation at 530 nm and longer wavelengths doesn't excite electronic states in the molecules, as is the case for other commonly used signal molecules like methylene blue.

Additional absorption features in the NIR (700-900 nm) (see Fig. S3a) are most likely a consequence of aggregation, as indicated by the lack of these features in the spectra of NPs in solution before deposition (see Fig. S4).

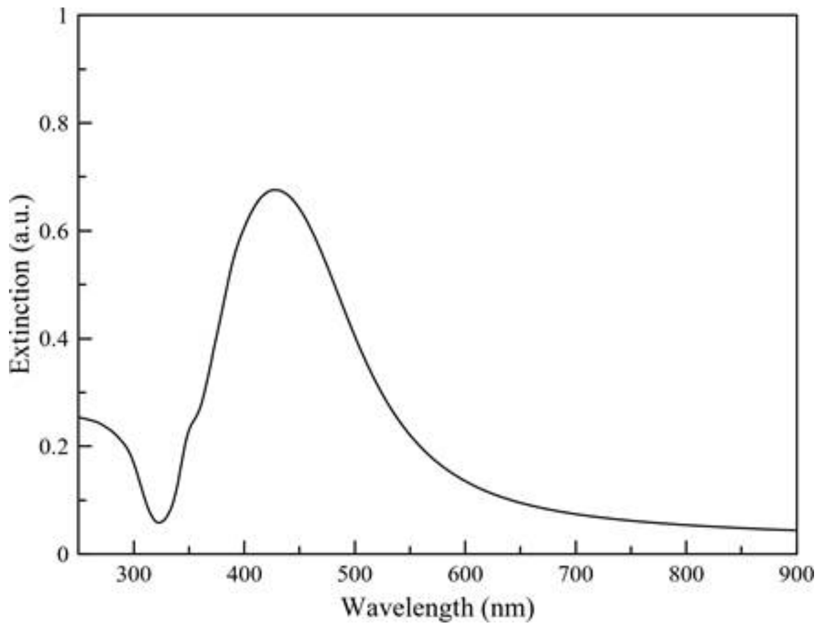

Figure S4: UV-Vis extinction of pure Ag NPs in solution before the deposition.

Section 2: Time-resolved IR spectroscopy:

Ultrashort laser pulses generated by a Ti:Sa CPA system (Coherent Libra) (central wavelength: 800 nm, pulse length: 120 fs, repetition rate: 1 kHz) were split up in a pump and probe beam path. In the pump path, the light was frequency doubled to 400 nm and used to amplify supercontinuum in a non-collinear optical parametric amplifier (NOPA, design by group of E. Riedle). The pump pulses are tunable between 500 and 700 nm with a pulse energy of up to 15 µJ. The pump is delayed by an optical delay line and the optical power is reduced by a half-wave plate before a polarizer.

The mid-infrared probe beam is generated by difference frequency generation (DFG) in an $AgGaS_2$ crystal of the signal and idler beams of a 2-stage collinear OPA. The probe beam is split into two beams called probe and reference. The pump, probe, and reference beams are focused on the sample by lens and off-axis parabolic mirrors. The probe spot size is below 50 µm and smaller than the pump beam (~150 µm) to ensure homogeneous excitation. Only the probe beam passes the excited region of the sample while the reference beam passes another spot. A 2x64 pixel MCT detector (Infrared Associates) placed after a grating spectrometer (Chromex 250is) detects the probe and reference beam and is read out for each single shot. The pump beam is chopped to half the repetition rate (500 Hz). Probe and reference pulses are divided into pump on and off and then each averaged. Absorbance change is calculated for probe and reference by $\Delta A_{probe\,;\,reference} = -\,log\,log\,(I_{on}^{probe\,;reference}\,/\,I_{off}^{probe\,;reference})$ ..

By - ΔA_{probe} - $\Delta A_{reference}$ the noise is greatly reduced, allowing measurement of photoinduced absorption signals as small as 10^{-4} OD.

The instrument response function is approx. 150 fs, estimated by the ultrafast signal rise in cesium lead bromide.

The probe spectrometer is calibrated by IR absorption of atmospheric gases (H_2O, CO_2) and polymer samples characterized by FTIR. The error in absolute frequencies is approx. 1 cm^{-1}, but the relative accuracy is higher. For measurements, the optical setup is purged with dry air.

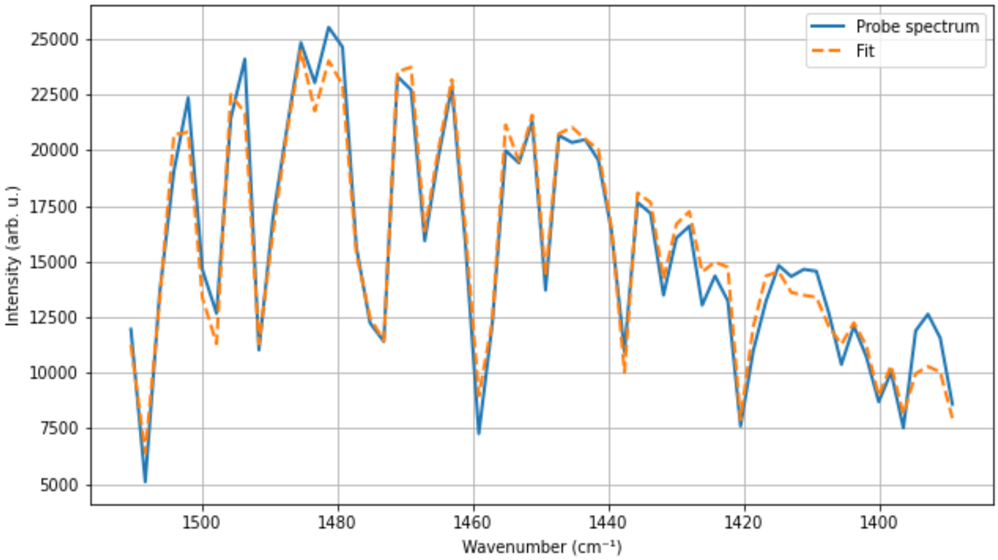

Figure S5: Fitting of the probe spectrum before purging (blue line) with a Gaussian function and the absorption of atmospheric water vapor obtained by FTIR (dashed orange line) to calibrate the spectrometer.

To ensure the stability of the samples and the optical setup during pump-probe measurements (delay scans), we regularly collected several scans in a row to reduce the correlation between delay and real-time effects.

Figure S6 shows TA spectra at a fixed delay for 3 consecutive scans on BPT-covered NPs (top figure), the corresponding time-traces (middle), and five time-traces of DDT-covered NPs at 2800 cm^{-1} (lower figure). As can be seen, no systematic change in amplitude or dynamics occurred, indicating the stability of the samples.

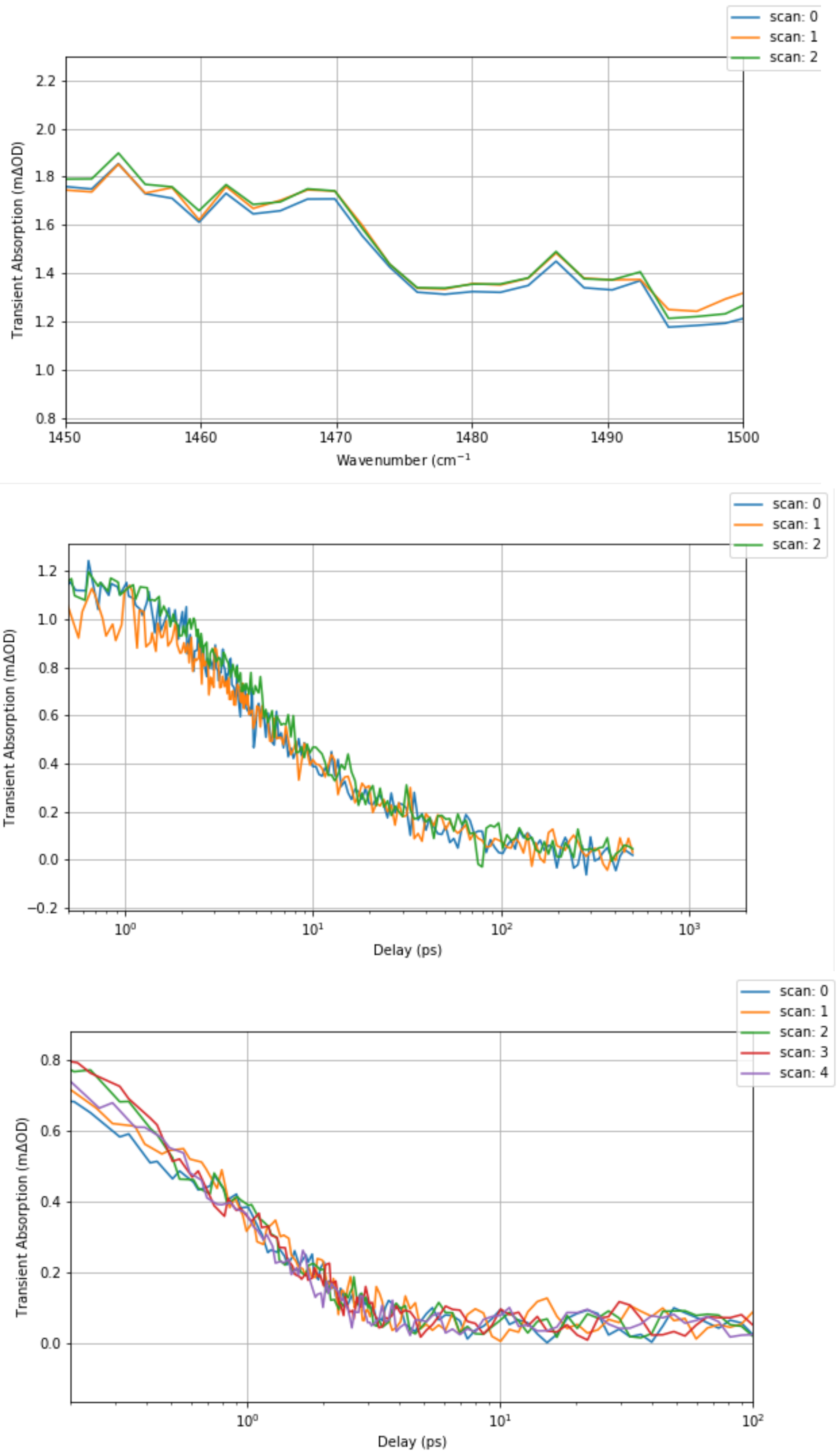

Figure S6 top: TA spectra of BPT, middle: delay traces for BPT, bottom: delay traces of DDT at 2800 cm^{-1} probe frequency.

Section 3: Probe-frequency dependent dynamics

3.1 Influence of spatial inhomogeneity

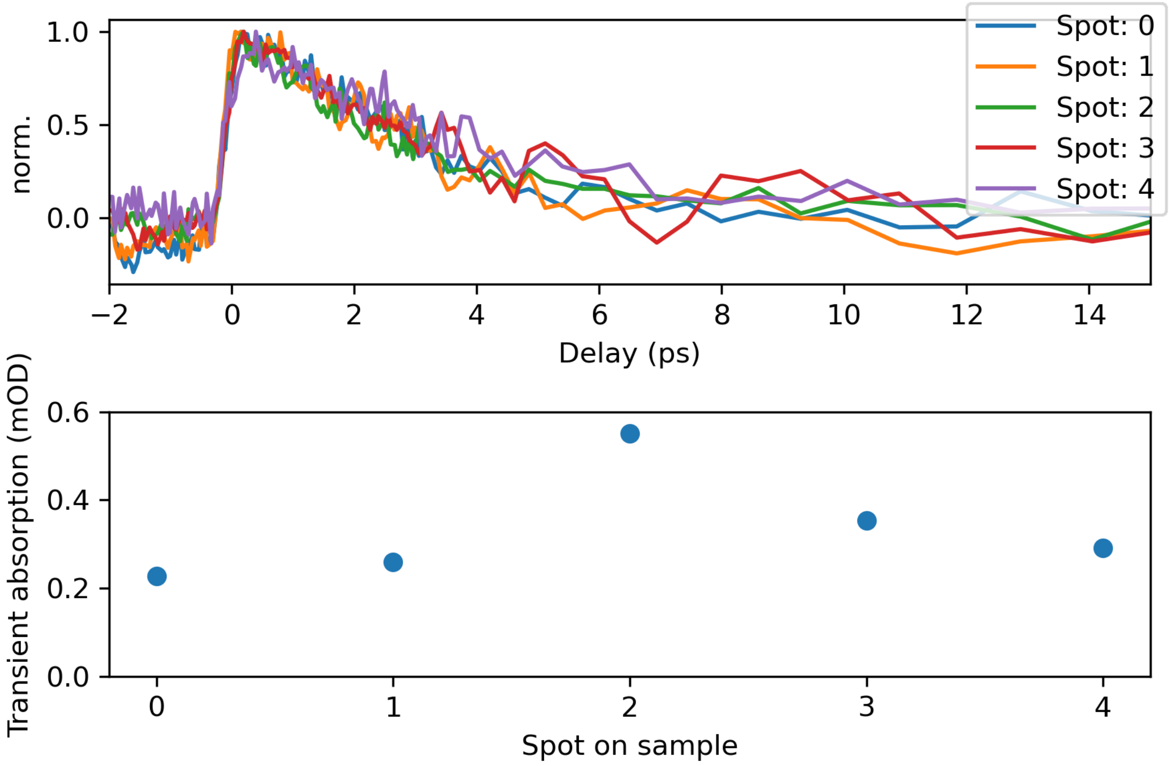

Figure S7: Transient absorption of DDT-covered Ag NPs at different spots on the sample for a probe frequency of 1500 cm^{-1}. The normalized transients (upper panel) demonstrate the uniform dynamic response of the entire sample. The lower panel shows the mean signal between 0.2 and 1 ps of the same dataset and the fluctuations in absolute amplitude. The TA signal strength depends directly on the local NP density.

3.2 Influence of pump fluence

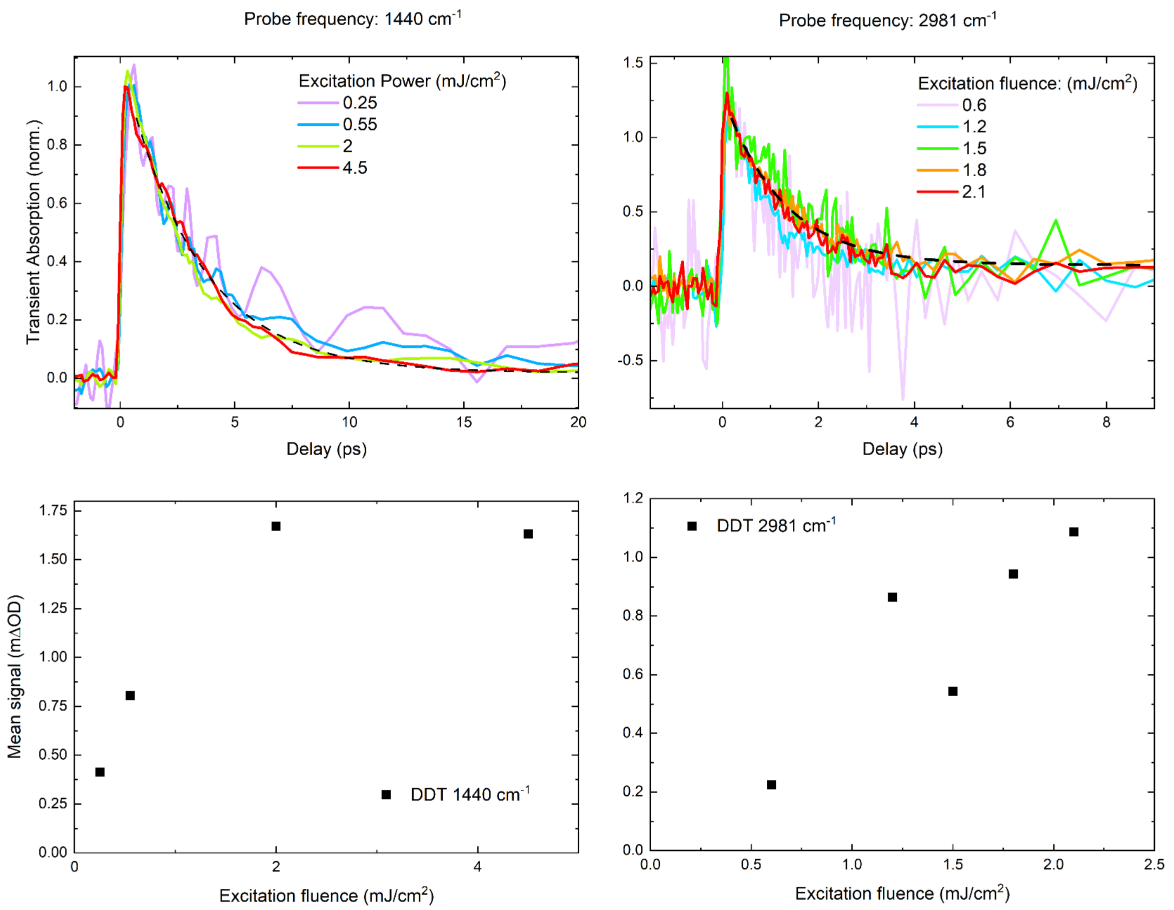

Figure S8: Fluence dependent TA traces for DDT-covered NPs at two probe frequencies. Within a certain range, the TA signal scales linearly with the pump fluence. Saturation of the signal is observed above approx. 2 mJ/cm^2 but does not affect the dynamics.

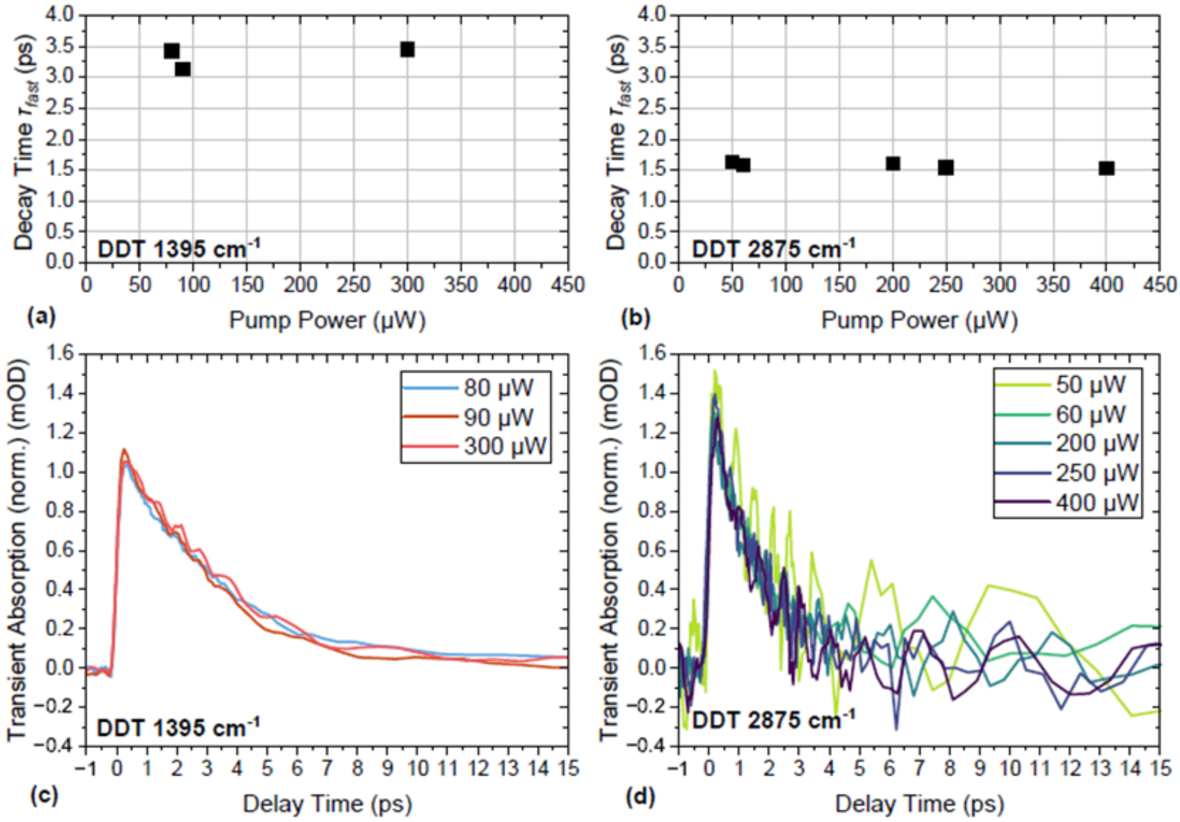

Figure S9: Excitation power and thus fluence-dependent dynamics (normalized transients in c and d, extracted time constants in a, b) for DDT at 2 probe frequencies. Laser power (µW) divided by repetition rate and spot size yields pump fluence.

(Multi)exponential fits are commonly applied to transients obtained in ultrafast spectroscopy. The underlying rate model for an exponential fit is based on a population N decaying according to $\frac{dN}{dt} = -kN$, where k is the decay rate. Deviations from this model, e.g. band-to-band or Auger recombination in semiconductors manifest themselves through higher orders of N in the decay rate (e.g. $\frac{dN}{dt} = -BN^2$). To find the most suitable model, we conducted fluence dependence measurements. By tuning the initially photogenerated population density, $N_{initial}$, but obtaining the same dynamics (see Figs. S8 and S9), we confirmed the validity of exponential fits. Further, we can state that the decay of the excited state is an intrinsic process, where interactions between multiple excited states or the associated quasiparticles are negligible.

To obtain the pulse energy, the pump power has to be divided by the repetition rate of 1 kHz. The fluence is calculated according to $F = \frac{4E_{pulse}}{d^2\pi}$. The laser spot size of ~120 µm was characterized by a beam cam (Ophir SP928). Still, the beam size is the parameter with the largest error due to changes in the beam size and shape between different experiments (alignment of NOPA). Thus, we obtain a fluence range of 0.5 - 4 mJ/cm^{-2}. Most experiments were conducted between 1-2 mJ/cm^{-2}. Laser damage was controlled by performing multiple delay scans for each measurement. Consecutive scans showed no sign of degradation. Laser-induced damage has never been observed.

3.3 Influence of probe frequency

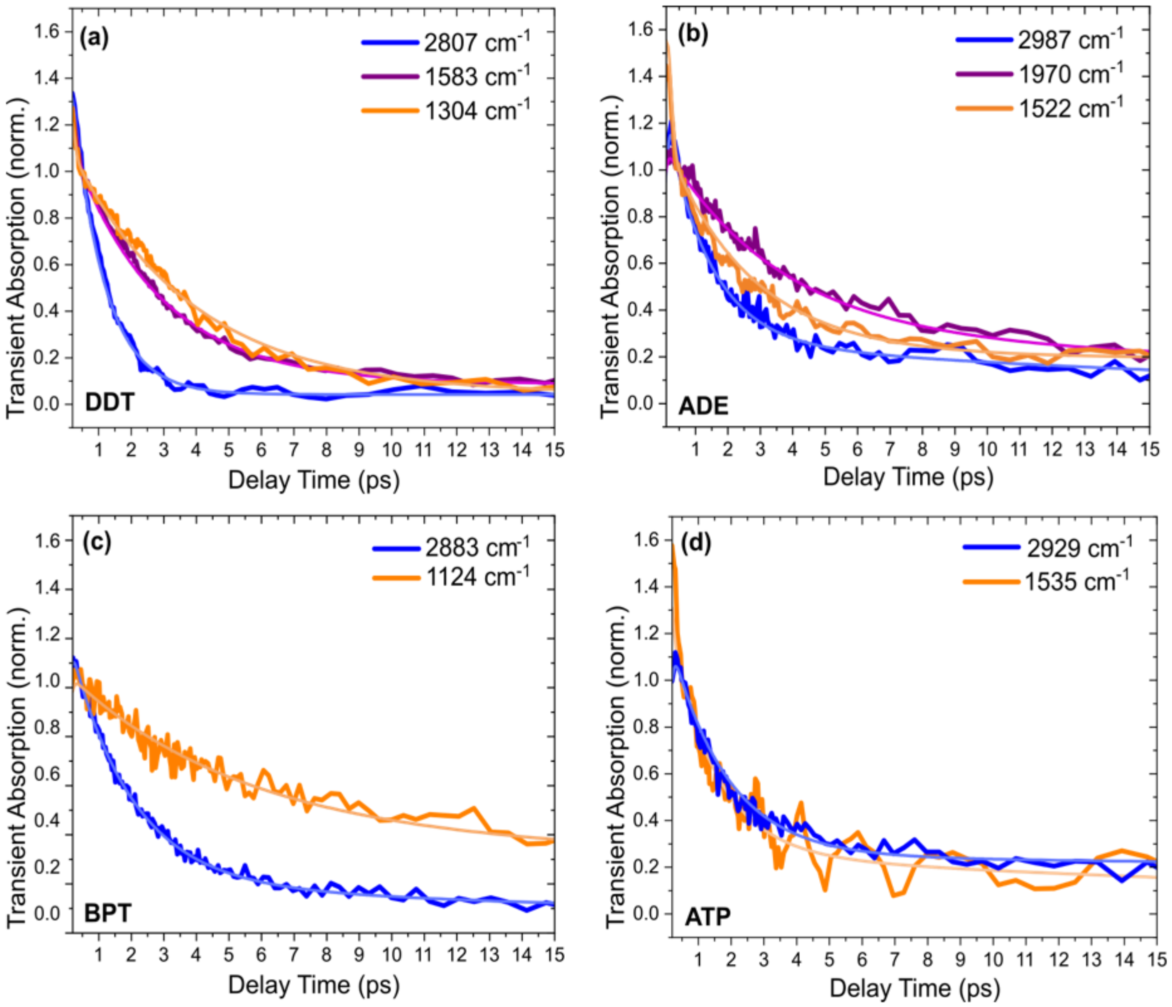

Figure S10: Transients of Ag NPs covered with DDT (a), ADE (b), BPT (c), and ATP (d) for different probe frequencies with monoexponential fits.

In Figure 2 in the main text, we report probe-frequency and molecule-specific decay constants. We support these findings by showing the (normalized) transients and fits in Figure S10. One can observe the probe-frequency-dependent dynamics for all samples but ATP.

3.4 Comparison mono-exponential and stretched-exponential

In disordered systems, stretched exponential fits often offer a better description of the dynamics than multi-exponential fits. The decay is described by :

$N(t) = e^{-(t/\tau)^{\beta}}$ with a stretching parameter β.

We compared mono-exponential fits and stretched exponential fits, each convoluted with a Gaussian injection term of width sigma, for time traces of DDT-covered NPs at two probe frequencies from -2 to 80 ps. Both fit functions allowed for an offset y0 at positive delays to account for small long-lived effects

1263 cm^{-1}:

Mono-exponential fit:

sigma = 0.12 ± 0.01 ps, tau = 3.4 ± 0.1ps, y0 = 0.02 ± 0.004

Stretched-exponential fit:

sigma = 0.1 ± 0.006, tau = 3.8 ± 0.1, beta = 1.13 ± 0.05, y0= 0.06 ± 0.008

2519 cm^{-1}:

Mono-exponential fit:

sigma = 0.01 ± 0.002 ps, tau = 1.34 ± 0.01 ps, y0= 0.05 ± 0.002

Stretched-exponential fit:

sigma = 0.01 ± 0.002, tau = 1.4 ± 0.02, beta = 1.06 ± 0.02, y0= 0.05 ± 0.002

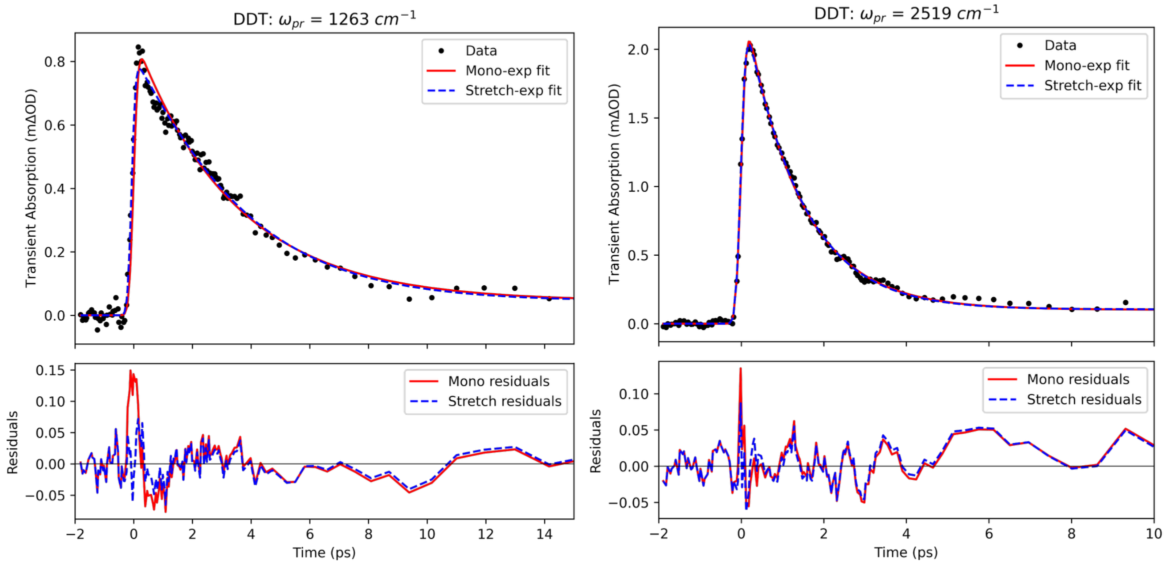

Figure S11: Comparison between mono-exponential (red) and stretched exponential (blue dashed) fits.

As can be seen in Fig. S11, the residuals are in close agreement, with the mono-exponential fit performing slightly worse. Importantly, the stretched fit optimized β to be close to zero, giving the special case of the ordinary mono-exponential decay. The improved fit quality of the stretched exponential comes at the cost that decay constants are arbitrary when beta is not considered. Therefore, we decided to use the simpler model of monoexponential decay. The fact that the stretching parameter is close to one may indicate that the system featuring these dynamics is not disordered, e.g. a disordered NP film with high order att the metal-molecule interface.

3.5 Data fitting and choice of model

To describe the probe-frequency dependent dynamics, we employed in total 3 different models: The Drude model with fixed τ_D (ATP), time-dependent τ_D (DDT, BPT) and a Polaron model (BPT, ADE). Prior to fitting, the zero-point (ZP) of all time-traces was shifted such that half of the maximum TA was reached at the ZP.
The large uncertainty of the amplitude was accounted for by a free scaling parameter between each experimental and model curve in the calculation of the residuals. The residuals (of multiple curves) were then minimized by the least squares fit provided by the Python scipy.optimize package.

In order to distinguish between different models, we employed the Akaike Information Criterion (AIC) and Bayesian Information Criterion (BIC).[4] Both models calculate a likelihood of the model being an accurate description based on the number of data points n, the number of free parameters k and the residual sum of squares RSS. The BIC is stricter regarding overparameterized models compared to AIC. In the special case of least-square fitting:

$$BIC = k \cdot ln(n) + n \cdot ln(\frac{RSS}{n}).$$

In general, if the difference in BIC between two models exceeds 10, then this is interpreted as strong evidence for the model with lower BIC.[4]

3.5.1 Time-dependent and constant τ_D

The time-dependent carrier density N(t) was described by the exponential decay with decay constant τ convoluted with a Gaussian representing the excitation/injection process with pulse duration σ:

$$N(t) = \Delta N\, e^{(\frac{\sigma^2}{2\tau^2}) - \frac{t}{\tau}} \cdot erf(\frac{\sigma}{\sqrt{2}\tau} - \frac{t}{\sqrt{2}\sigma}).$$

The evolution of the Drude collision time, e.g. by many-body renormalization and hot carrier cooling or solvation, was described as:

$$\tau_D(t) = 0.5\Delta\tau_D \cdot erf(-\frac{t}{\sigma}) \cdot (1 - e^{-\frac{t}{t_{\tau D}}}) + \tau_{D,0}$$

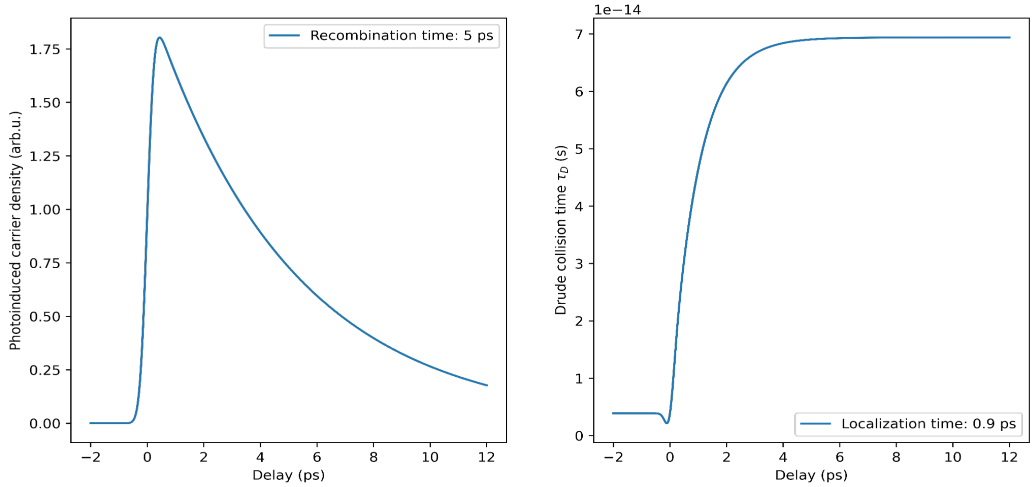

Figure S12: Modeling of the photoinjected carrier density and time-dependent Drude collision time.

Here, we define ΔBIC = BIC(variable τ_D) - BIC (constant τ_D)

Table S1: Fit parameters of the model preferred by ΔBIC

Molecule	$\Delta\tau_D$ (fs)	$t_{\tau D}$ (ps)	τ_{recomb} (ps)	ΔBIC
DDT	(19±6)	1.1	5.6	-770
BPT	(13±1)	3.4	30	-1260
ATP	—	—	(1.5±0.1)	110

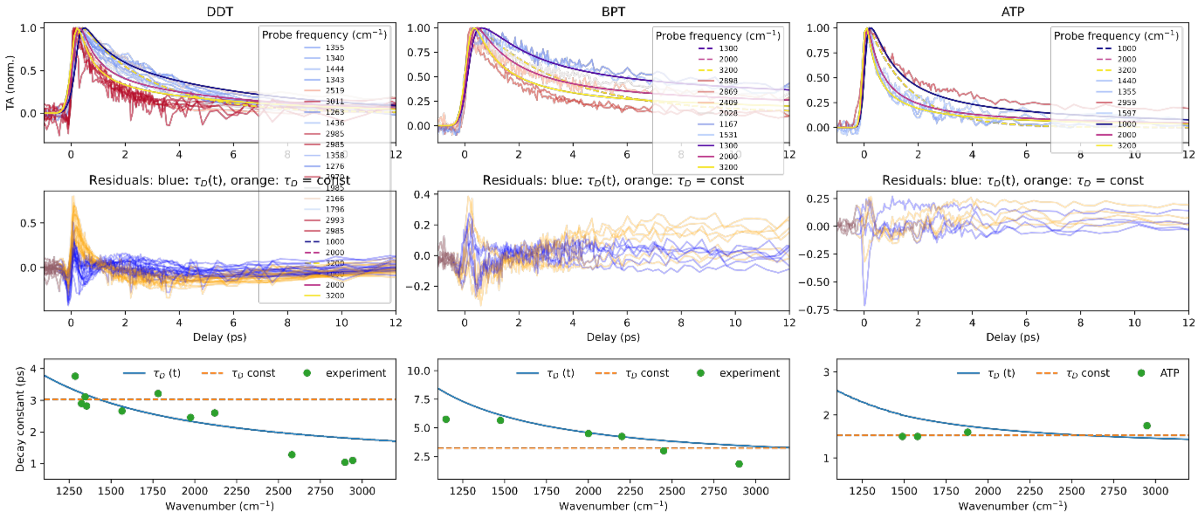

Figure S13: Top panel: all fitted data with color coding for the probe frequency and time traces predicted by the models. Dashed lines correspond to the constant tau model. Middle panel: Fitting residuals of each measured curve for the variable (orange) and constant (blue) τ_D models. Lower panel: Time constants by monoexponential fitting for data (dots) and for the time-traces calculated with the models with optimized parameters.

3.5.2 Time-dependent τ_D vs. Polaron model

The goal was to model mIR absorption spectra for a population of free carriers transitioning to a population of polarons (or other bound states) while decaying. The model calculates the time and frequency-dependent absorption by a linear combination of the Drude and polaron spectra with a parameter p(t):

$$A(t, \omega) = p(t)A_{pol}(\omega)N(t) + [1-p(t)]\, A_{Drude}(\omega)$$

The $A_{Drude}(\omega)$ term already contains the carrier recombination N(t) via the plasma frequency. The Drude collision time (and effective mass) is kept constant in this model.

p(t) describes the transition from a free carrier to a polaron and was modeled similar as $\tau_D(t)$ with a polaron formation time t_{pol}: $p(t) = 0.5 \cdot erf(-\frac{t}{\sigma}) \cdot (1 - e^{-\frac{t}{t_{pol}}})$

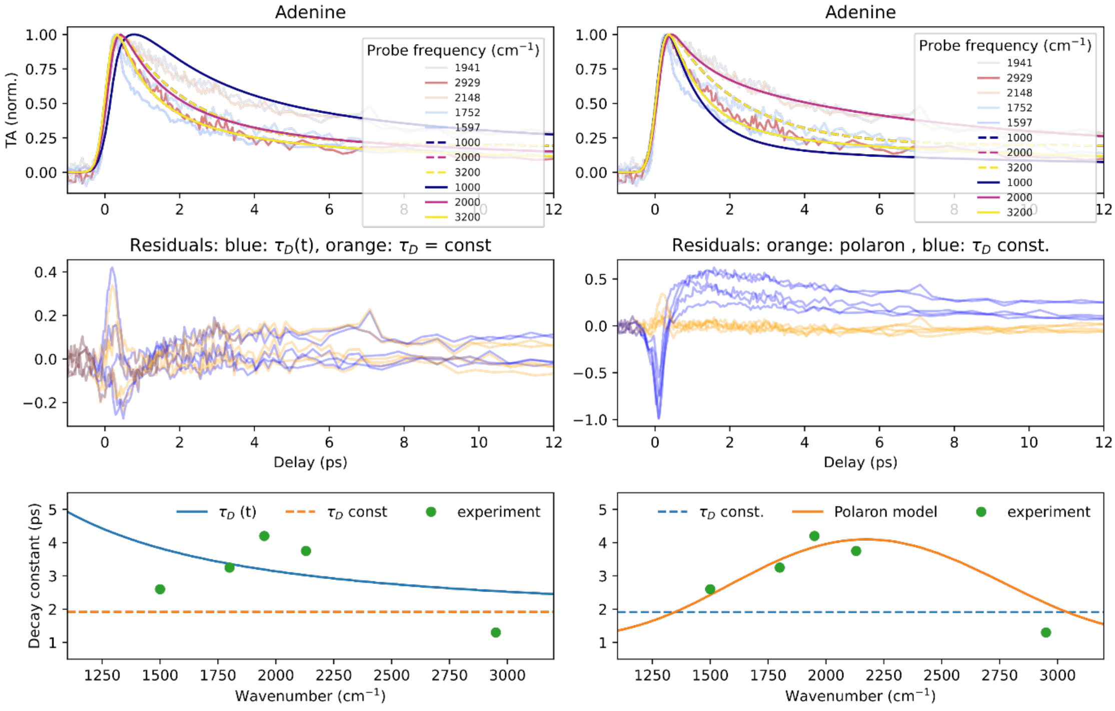

Figure S14: top: Experimental time-traces with time-traces of the models, middle: residuals of the models, bottom: decay constants by exponential fitting for the models and experimental data.

Figure S14 shows a comparison between constant and time-dependent Drude collision time models (left side) and between constant Drude collision time and the small polaron model (right side). The ΔBIC = BIC(variable τ_D) - BIC (constant τ_D) is 120, favoring the constant τ_D model and ΔBIC = BIC(polaron) - BIC (constant τ_D) is -1120, meaning the polaron model is very likely to be a more accurate description of our data.

The optimized parameters for the polaron model are a binding energy of (0.10±0.02) eV, a polaron formation time of 1 ps and a recombination time of 6.8 ps.

In addition, we performed a sensitivity analysis to estimate the impact of limited spectral range (both high and low frequency side) on the fitting procedure. We generated artificial data (time-traces) in the measured frequency range and added 10% noise (relative to the strongest signal). Then we determined the polaron binding energy for several energies of the model that generated the data.

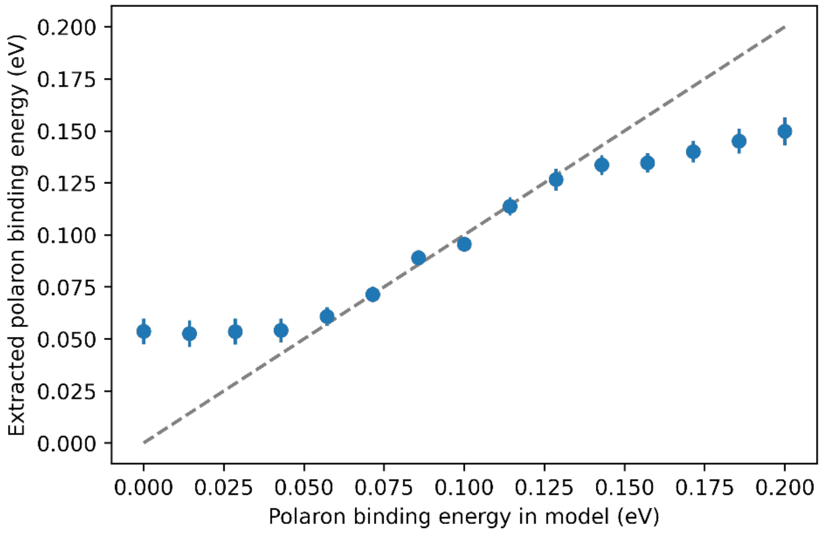

Figure S15: Comparison of extracted polaron binding energy versus model input

As can be seen in Figure S15, the extracted polaron binding energy matches the model calculation when the peak of the polaron absorption at the binding energy lies within the measured interval. For the case of Adenine-covered NPs with an extracted binding energy of (0.10±0.02) eV, the spectral window studied by us seems to be sufficient.

3.6 Control experiments with NIR pump:
To ensure that the reported transient effects are a consequence of the LSPR excitation, we performed additional measurements with a nIR pump at 800nm /1.55 eV (Fig. S16). We observed no change in absorption under similar excitation conditions and weak, long-lived negative TA (compare for REF in Fig. 1d) under stronger pumping.

In summary, the main findings of this study were only observed for excitation of the LSPR at 400-600 nm.

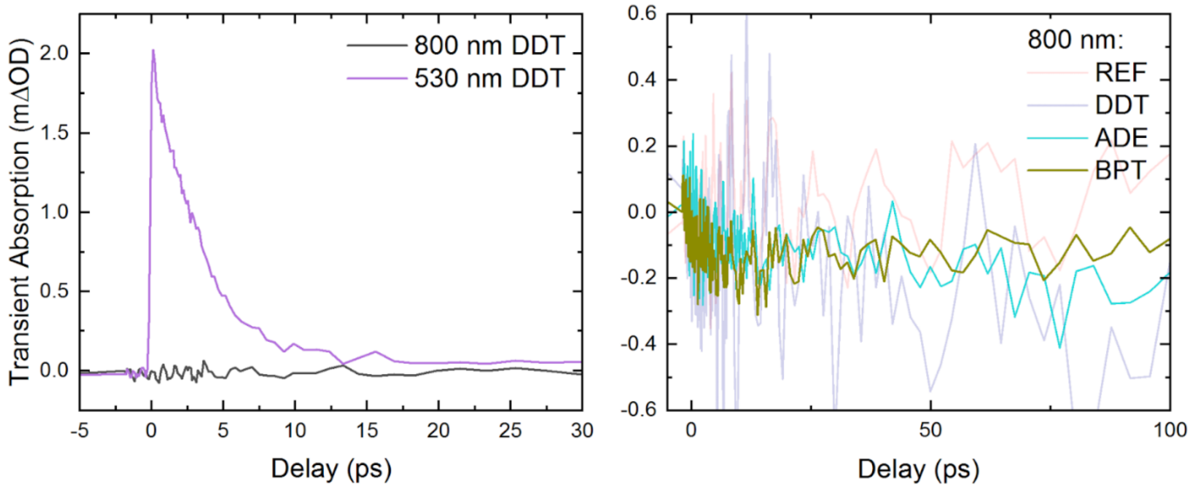

Figure S16 shows transient absorption of DDT-covered NPs at a probe frequency of 1350 cm-1 for excitation at 530 and 800 nm with similar pump fluence (0.6 mJ/cm^2). No TA is observed for 530 nm. Under stronger pump fluence (3 mJ/cm^2), we observed a weak negative TA signal with dynamics that are not sensitive to the adsorbed molecular species.

Section 4: Thermal changes in IR absorption and thermal interfacial conductance

4.1 Extracting the dynamics of adsorbate temperature

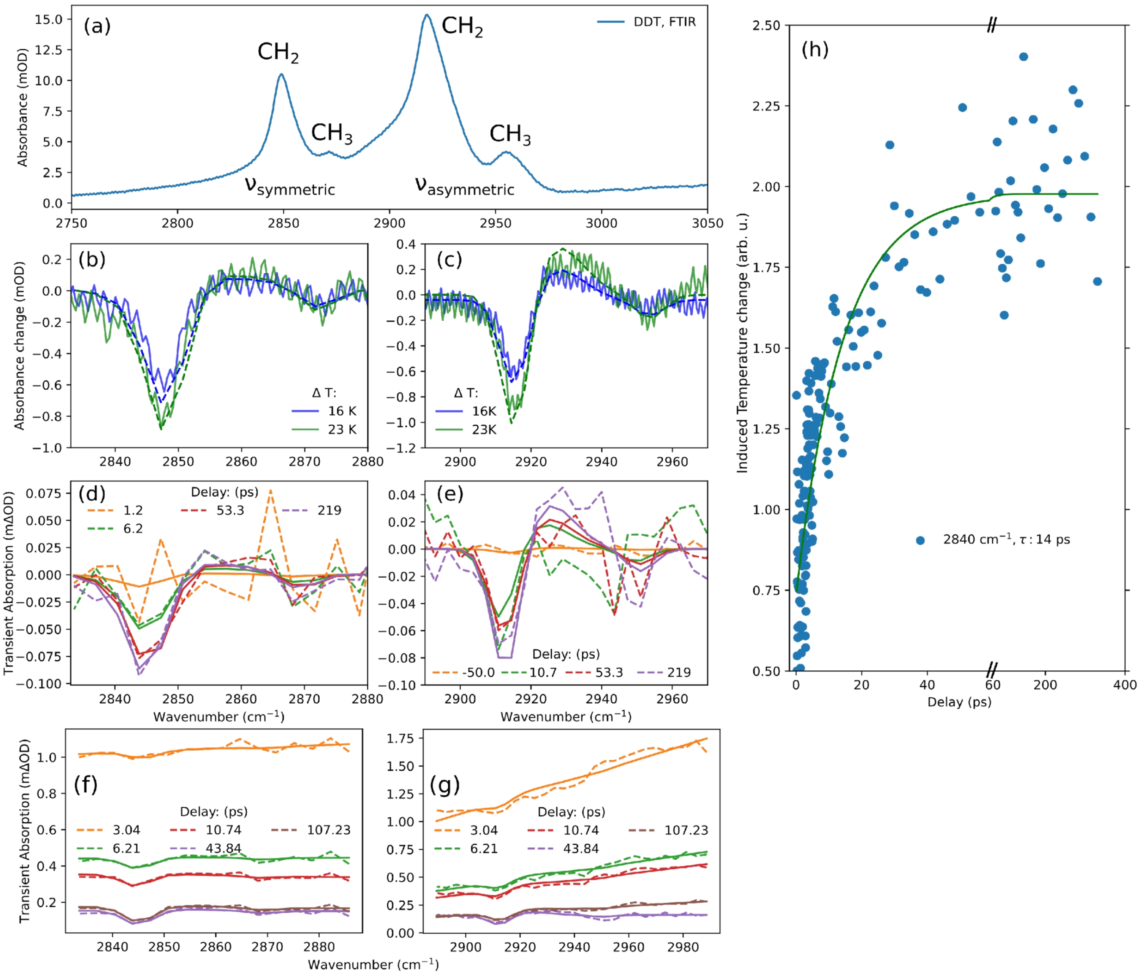

Figure S17. a, IR absorption of DDT-covered Ag NPs. b, c, Temperature-induced changes in IR absorption (solid lines) with empirical function (dashed lines). d, e, TA spectra with subtracted background (dashed line) along with fits (solid lines) for selected pump-probe delays. f,g, same w.o. background subtraction, h, Time-resolved change in sample temperature (via proxy) determined by fits in Figs. 2 d,e (dots) with exponential fit (solid line).

Besides the sensitivity to free charge carriers, the mid-IR probe pulse is absorbed by molecular vibrations, which are sensitive to the local charge carrier density and temperature. To determine the increase in adsorbate temperature after optical excitation, we have chosen aliphatic C-H stretching modes, which are most pronounced for DDT molecules due to their composition (see Fig. 1b, main text). The bonds responsible for this IR absorption are homogeneously distributed along the DDT molecule. C-H stretching modes are generally less sensitive to local charges than IR modes between 1000-2000 cm^{-1} or N-H stretching modes. To our knowledge, there is no report of photoinduced IR absorption at the C-H stretching frequencies by charge carriers. Therefore, we employ these IR modes as temperature-selective local reporters.

Figure S17a shows the IR spectrum of DDT-covered Ag NPs. Compared to pure DDT, the aliphatic C-H stretching modes are slightly red-shifted by 7-10 $cm^{-1,}$ as previously reported.[5] We heated the samples inside the FTIR and obtained the temperature-induced absorbance

changes shown in Figs. S17b and c. The absorbance change was modeled by an empirical function containing several Gaussian peaks of different sign, amplitude, and width (see dashed lines in Figs. S17 b and c). The function is then scaled with a single amplitude and fitted to the temperature-dependent data to obtain the conversion factor between amplitude and temperature change. Lastly, the function, modified with a linear term to account for the broad electronic absorption, has been fitted to TA spectra shown in Figs. S17d and e (broad electronic background subtracted, raw data shown in f and g). In the time-resolved data (Figs. S17d and e), one observes bleaching at the same central frequency as in temperature-dependent IR absorption, evolving over several ps. The extracted dynamics of the temperature proxy is shown in Fig. S17h and is characterized by a slow increase and a plateau that persists through the entire delay range we studied (400 ps).

In principle, the heat transfer to the molecule is convoluted with the intramolecular vibrational relaxation. Previous studies have observed a time constant of 0.4 ps per CH_2 unit, meaning the vibrational relaxation through the entire chain of DDT takes less than five ps.[6] Thus, the time constant of 14 ps, determined by monoexponential fitting, is dominated by the heat transfer to the molecule and is in good agreement with recent experimental and theoretical work.[7] In principle, one would expect a later onset for the bleaching of the CH_3 stretching vibrations, but these signals are too small for the extraction of dynamics. The plateau at long time delays is expected as heat diffusion to the substrate occurs on the slower nano-to-microsecond timescale.

We want to emphasize that determining the absolute value of the temperature increase in the adsorbate layer is a difficult task and beyond the scope of this work. The by far largest uncertainty stems from DDT molecules attached to silver NPs, which, because of their size or position (e.g. in the shadow of other NPs), haven't been excited by the optical pump pulse. The DDT molecules attached to these 'cold spots' are heated along with the entire sample in the calibration measurement shown in Figs. S17b and S17c, and contribute to the $\Delta A(\Delta T)$ but do not contribute to the light/pump-induced signal. This reduces the apparent temperature change by an unknown factor. The extracted dynamics are not affected by this issue since molecules that are not excited do not contribute to the transient signal. Note that in SERS measurements, one often is faced with the opposite effect because there the vibrational signal is a weighted average, favoring spots with high field enhancement.

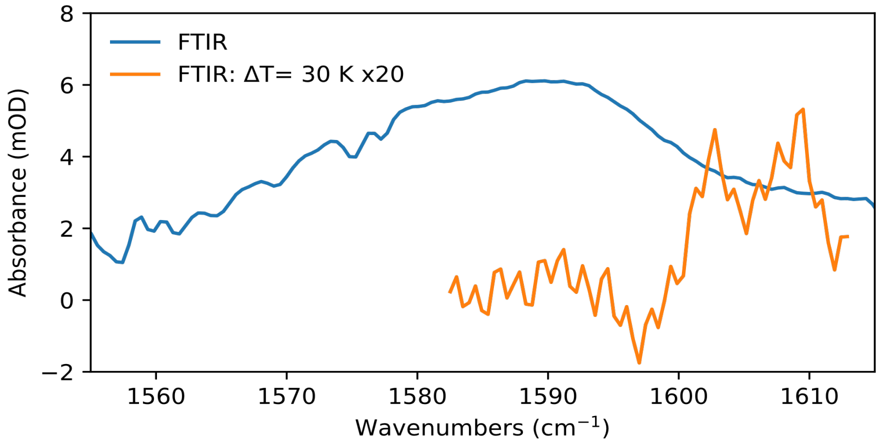

Figure S18: Temperature-induced absorbance change in ATP NPs. Range reduced due to water vapor artefact. Ultrafast bleaching in pump-probe observed at 1585 cm^{-1}. The temperature-induced signal is weaker compared to the other samples and not observed in the TA spectra shown in Fig. 3.

4.2 Calculation of interfacial conductance

We used a simple model to relate the rise time of thermal signatures to the interfacial conductance G and heat capacity. The underlying assumptions are that the lattice temperature of the NPs T_{NP} stays constant throughout the heat transfer because the total heat capacity of the NPs, C_{NP} >> C_{ML}, the heat capacity of the adsorbant layer (for NPs > 10 nm). Further, we neglect heat transfer from the adsorbate layer to the environment, based on the plateau of the thermal signal that we observe for 100s of picoseconds.

The temperature of the adsorbate layer T_{ML} is described by:

$$\frac{dT_{ML}}{dt} = \frac{Q}{C_{ML}} = \frac{GA}{C_{ML}}(T_{NP} - T_{ML}) \rightarrow T_{ML}(t) = T_{NP}(1 - e^{-t/\tau});\ \tau = \frac{C_{ML}}{GA}$$

with the molar heat capacity c_m, area per molecule a_m, and Avogadro's number N_A we obtain $G = \frac{c_m}{N_A a_m \tau}$.

Table S2: Fundamental time constants derived from experimental data and calculated values (by use of literature values) for thermal conductivity across the NP-molecule interface.

Adsorbant molecule	Rise time of thermal signal (ps)	molar heat capacity (J K^{-1} mol^{-1})	area per molecule (m^2)	Estimated conductance G (MW m^{-2} K^{-1})	NP-Adsorbate coupling G_{mol} (pW K^{-1})
Adenine	(1.5 ± 0.1)	145[8]	~4.7 $\cdot 10^{-19}$ (ref. [9])	340	(160 ± 10)
4-biphenylthiol	(4.4 ± 0.3)	198[10]	~3.8 $\cdot 10^{-19}$ (ref. [11])	200	(75 ± 5)
1-dodecanethiol	(14 ± 1.5)	443[12]	~2.2 $\cdot 10^{-19}$ (ref. [13])	230	(55 ± 6)

The calculated conductance G of DDT is in good agreement with previous studies on thiolated alkane chains (220 (±100) MW m^{-2} K^{-1}).[13] Please note that the error is dominated by the uncertainty in surface coverage and molecule density. The area per molecule has been calculated according to the unit cells observed via STM/AFM measurements and doesn't account for curvature or incomplete coverage, arising in the NP film.

Without considering the interfacial conductance per area, which depends strongly on the areal density of the molecules, we can compare the per-molecule conductivity by only comparing the molecular heat capacity with the rise time. From this, it follows that Adenine vibrations in adenine couple strongest to the phonons of the silver NP, followed by BPT and DDT.

Section 5: Energy alignment of molecules with silver

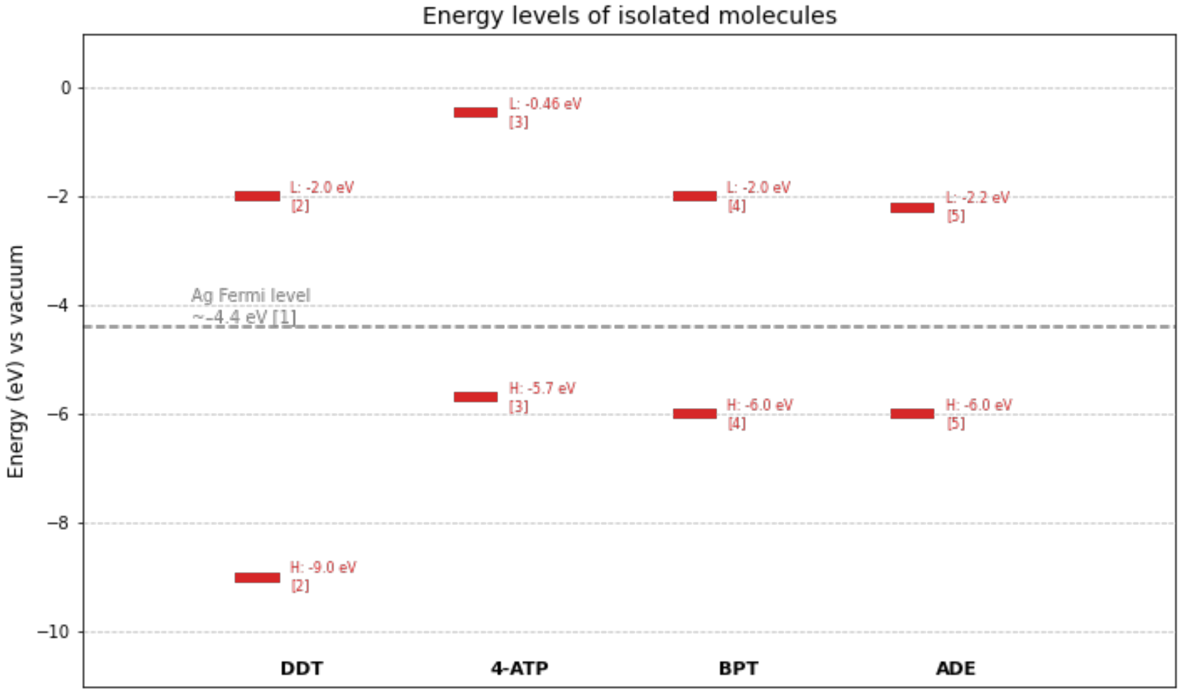

Figure S19: Alignment of HOMO-LUMO levels relative to the Fermi level of silver.

In the main manuscript, charge transfer and the optical response of free charge carriers is discussed. As is well established in the semiconductor community, Drude and Polaron absorption occur for both electrons and holes.[14] The presence of photoinduced IR absorption does not hint at the direction of electron transfer. To address this ambiguity in a first step, we look at the energy barriers of electron transfer from Ag into the LUMO levels and from HOMO levels towards Ag (see Fig. S19).

For all molecules except DDT, the energy alignment indicates electron transfer from the HOMO of the molecules towards holes below the Fermi level of silver (E_F) after plasmon excitation. Note that the energy alignment can shift due to hybridization of the adsorbate layer with the silver NP.[15-16] This effect typically shifts the HOMO level up, further reducing the E_{HOMO} - E_F gap.

Section 6: Non-thermal (ultrafast) vibrational spectra:

Calculation of vibrational spectra:

IR spectra were computed with the aid of the ORCA quantum chemical calculation package by Neese et al.[17] The geometry for each molecule and its ions was optimized in a first step at the density functional theory (DFT) level using the def2-SVP basis set. The IR spectra were computed with the BP86 exchange-correlation functional and def2-TZVP basis set, a combination that is generally recommended for such calculations with a good balance between computational efficiency and vibrational frequency accuracy. Where possible, computed molecular spectra were compared with measured IR spectra. A good correspondence was obtained for all spectra which provided confidence in the approach adopted.

ATP:

Calculated IR peaks have been shifted by 23 cm^{-1} to match the FTIR data.

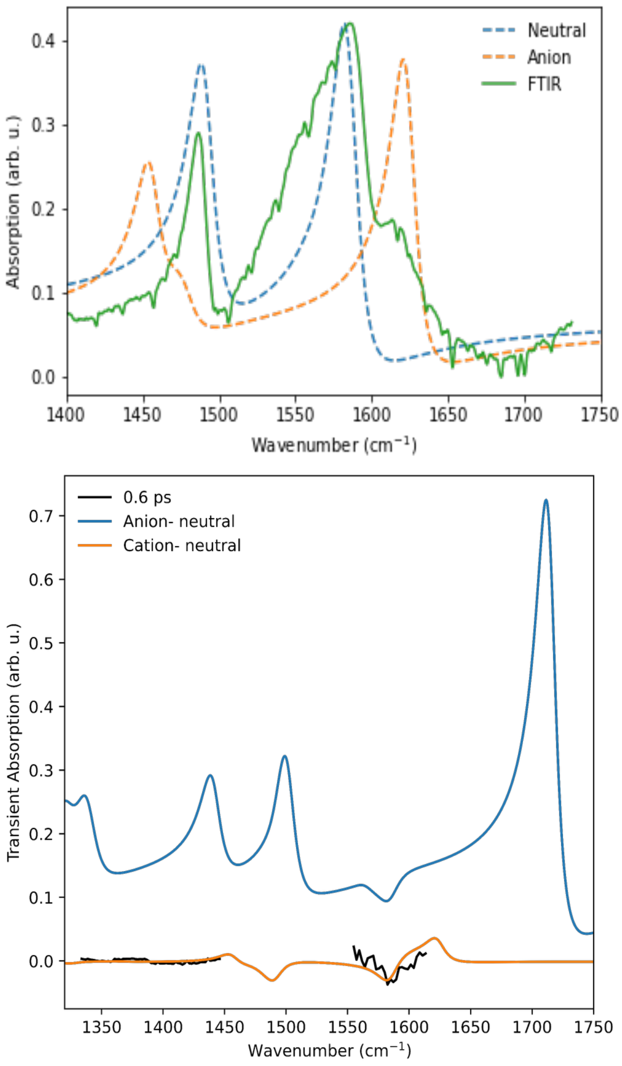

Figure S20 top: Comparison between FTIR and the calculated spectra of the neutral and anion ATP molecules.

The experimental data are strongly in favor of the electron transfer towards the Ag NPs. In the main paper, we analyze the dynamics of the mode at 1580 cm^{-1}, but this is ambiguous. The calculated spectrum for the anion features another strong absorption at 1440 cm^{-1}, which is not observed in TA. The broad background in TA spectra was subtracted to focus on the vibrations.

BPT:

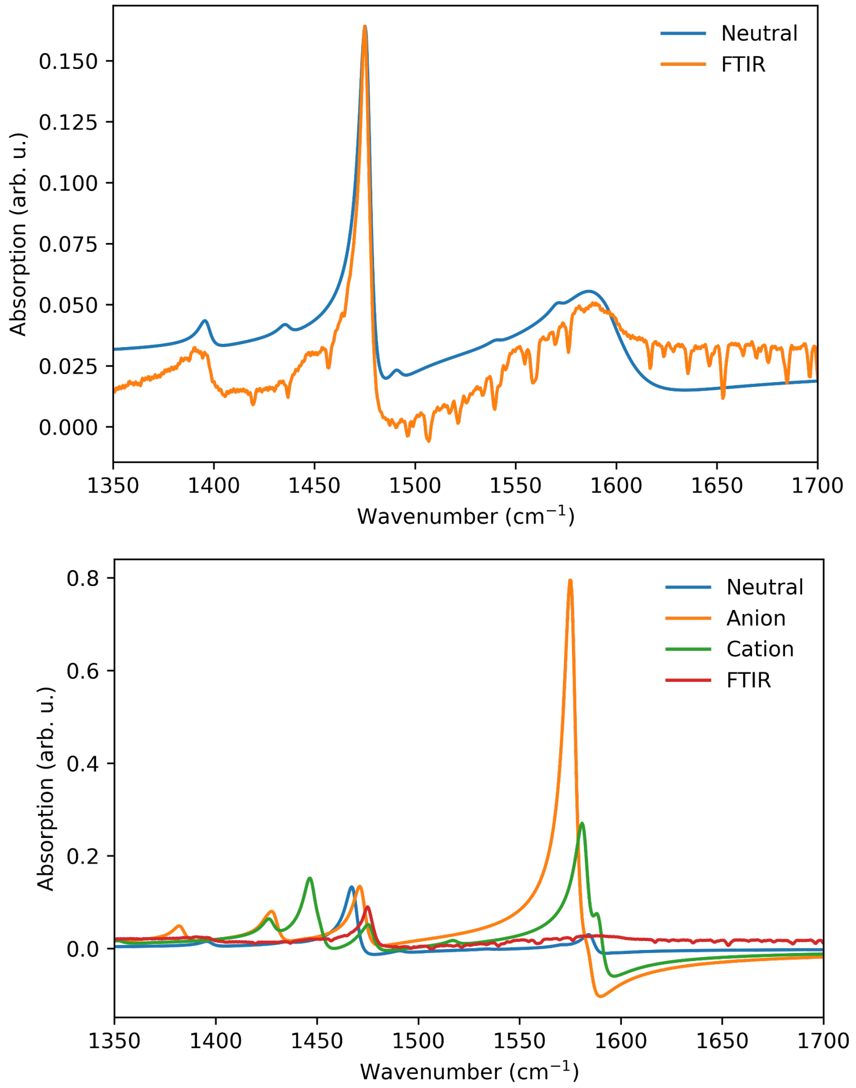

Figure S21 top: Comparison between FTIR and calculated IR spectrum of neutral BPT with IR peaks shifted by 8 cm^{-1}. bottom: Calculated IR spectra for charged BPT molecules, in agreement with previous theoretical work.[18]

As explained in the main manuscript in the subsection ‘Vibrational response’, the non-thermal lineshape of BPT features a negative modulation of the free carrier absorption at a frequency with negligible ground state absorption, eliminating bleaching as an explanation. Recent work has found similar features in TA spectra and linked them to an anti-Fano resonance of a charge transfer state.[19] We expect that the IR spectrum of the charge transfer state differs from the predicted spectrum of the charged molecules in vacuum and have not observed the

vibrational modes at 1580 cm^{-1}, which were calculated for positively and negatively charged BPT molecules in vacuum. Note that a theoretical treatment of the charge transfer state is beyond the scope of this work.

ADE:

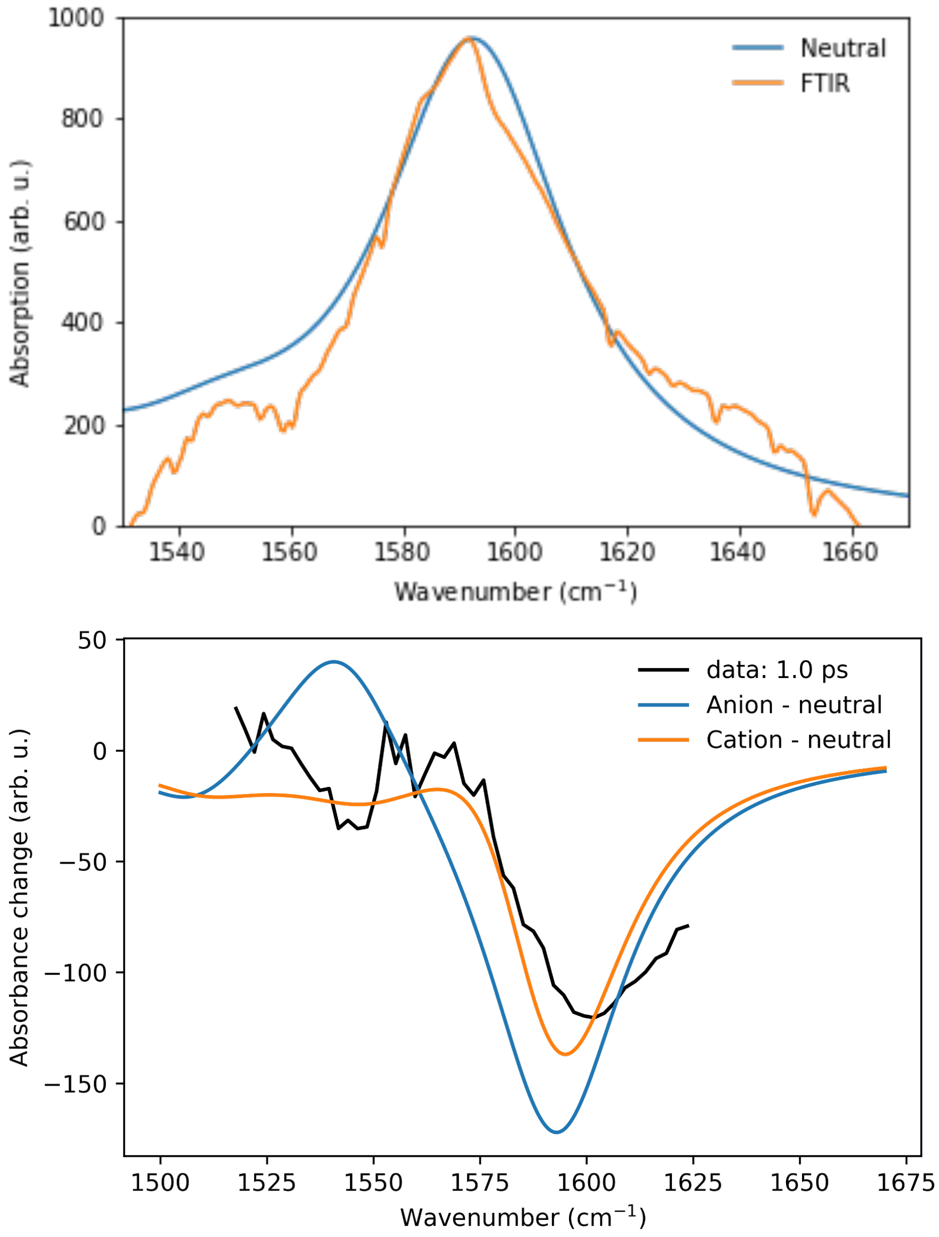

Figure S22 top: Comparison between FTIR and calculated IR spectrum of neutral adenine, spectra shifted by 25 cm^{-1}. bottom: Comparison between calculated absorbance changes and experimental data. The lack of induced absorption at 1530 cm^{-1} indicates electron transfer towards the Ag, leaving the Adenine in a cation state.

Section 7: Role of adsorbed water

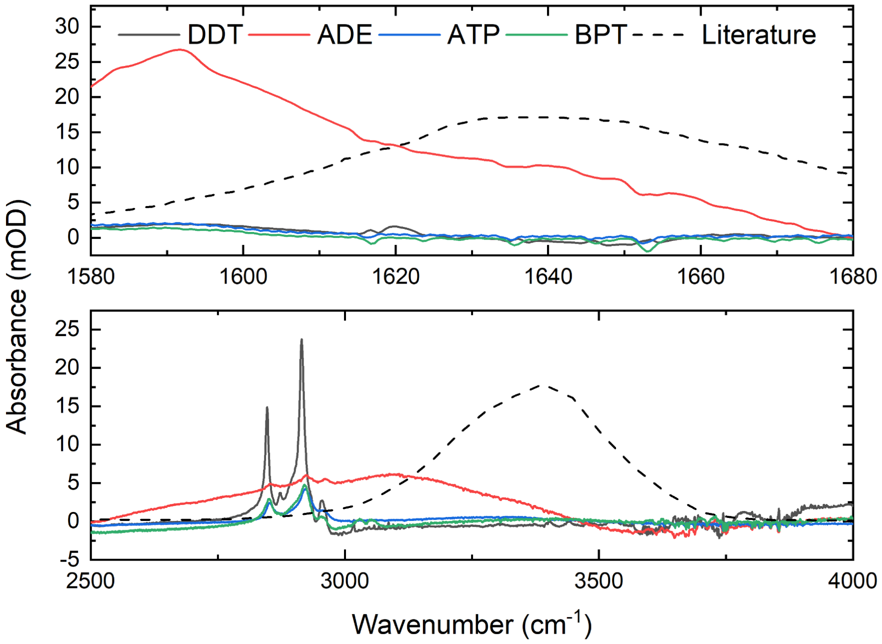

Figure S23: FTIR of all samples and liquid water (dashed lines) for comparison.

The abundance of water in the atmosphere and its tendency to cover metallic surfaces raises the question of whether water is present in the studied samples and how it influences the carrier dynamics.

Liquid water features two main IR peaks at ~1640 cm^{-1} (bending mode) and 3400 cm^{-1} (O-H stretching). Figure S23 shows all samples in the respective regions compared to literature water spectra [20-21] scaled for clarity. In both panels, only the spectra of ADE show similarity to the presence of water, as a shoulder appears at 1640 cm^{-1}. If the peak around 1640 cm^{-1} is assigned to water, one would expect another peak at 3400 cm^{-1} with an amplitude that is four times larger.[22] This is not observed, and the peaks at 1640 cm^{-1} and 3100 cm^{-1} are in line with previously described IR spectra of adenine.[23]

Nevertheless, the expected IR absorption for a single mono-layer, potentially enough to participate in solvation, is on the order of only 0.05 mOD and 0.2 mOD for the peaks at 1640 cm^{-1} and 3400 cm^{-1}, respectively. Therefore, our experimental data are not sufficient to exclude the presence of single monolayers of water. Because of the large spectral width and the known shifting in the center positions of water IR modes, even noise-free data couldn't answer this question unequivocally.

We want to emphasize that the main observation of this paper, the molecule-specific shift in the photoinduced broad IR absorption throughout few picoseconds, is highly relevant and unexpected. If water were the only reason for solvation, we would expect the binding energy and resonant energies to be in the near-infrared and expect the same results for the reference sample of pure Ag NPs; in contradiction to our data. The comparison with literature is not affected, as most time-resolved IR or SERS studies on plasmonic NPs have been conducted under atmospheric conditions.

References

1. Lee, P. C.; Meisel, D., Adsorption and surface-enhanced Raman of dyes on silver and gold sols. *The Journal of Physical Chemistry* **1982,** *86* (17), 3391-3395.
2. Botiz, I.; Codescu, M.-A.; Farcau, C.; Leordean, C.; Astilean, S.; Silva, C.; Stingelin, N., Convective self-assembly of π-conjugated oligomers and polymers. *Journal of Materials Chemistry C* **2017,** *5* (10), 2513-2518.
3. Stefancu, A.; Nan, L.; Zhu, L.; Chiș, V.; Bald, I.; Liu, M.; Leopold, N.; Maier, S. A.; Cortes, E., Controlling Plasmonic Chemistry Pathways through Specific Ion Effects. *Advanced Optical Materials* **2022,** *10* (14), 2200397.
4. Kass, R. E.; Raftery, A. E., Bayes Factors. *Journal of the American Statistical Association* **1995,** *90* (430), 773-795.
5. Jayabharathi, J.; Sundari, G. A.; Thanikachalam, V.; Jeeva, P.; Panimozhi, S., A dodecanethiol-functionalized Ag nanoparticle-modified ITO anode for efficient performance of organic light-emitting devices. *RSC Advances* **2017,** *7* (62), 38923-38934.
6. Wang, Z.; Pakoulev, A.; Dlott, D. D., Watching Vibrational Energy Transfer in Liquids with Atomic Spatial Resolution. *Science* **2002,** *296* (5576), 2201-2203.
7. Mertens, J. C. J.; Spitzbarth, B.; Eelkema, R.; Hunger, J.; van der Veen, M. A., Predicting and Probing the Local Temperature Rise Around Plasmonic Core–Shell Nanoparticles to Study Thermally Activated Processes. *ChemPlusChem* **2024,** *89* (9), e202400134.
8. Emel'yanenko, V. N.; Zaitsau, D. H.; Shoifet, E.; Meurer, F.; Verevkin, S. P.; Schick, C.; Held, C., Benchmark Thermochemistry for Biologically Relevant Adenine and Cytosine. A Combined Experimental and Theoretical Study. *The Journal of Physical Chemistry A* **2015,** *119* (37), 9680-9691.
9. Perdigão, L. M. A.; Staniec, P. A.; Champness, N. R.; Kelly, R. E. A.; Kantorovich, L. N.; Beton, P. H., Experimental and theoretical identification of adenine monolayers on Ag-terminated Si(111). *Physical Review B* **2006,** *73* (19), 195423.
10. Saito, K.; Atake, T.; Chihara, H., Incommensurate Phase Transitions and Anomalous Lattice Heat Capacities of Biphenyl. *Bulletin of the Chemical Society of Japan* **2006,** *61* (3), 679-688.
11. Matei, D. G.; Muzik, H.; Gölzhäuser, A.; Turchanin, A., Structural Investigation of 1,1′-Biphenyl-4-thiol Self-Assembled Monolayers on Au(111) by Scanning Tunneling Microscopy and Low-Energy Electron Diffraction. *Langmuir* **2012,** *28* (39), 13905-13911.
12. Tutubalina, V. P. G., F.G.; Konyukhova, T.M., Study of the heat capacity of n-sulfides. *Teplo-Massoobmen Khim* **1982**.
13. Wang, Z.; Carter, J. A.; Lagutchev, A.; Koh, Y. K.; Seong, N.-H.; Cahill, D. G.; Dlott, D. D., Ultrafast Flash Thermal Conductance of Molecular Chains. *Science* **2007,** *317* (5839), 787-790.
14. Salomon, A.; Cahen, D.; Lindsay, S.; Tomfohr, J.; Engelkes, V. B.; Frisbie, C. D., Comparison of Electronic Transport Measurements on Organic Molecules. *Advanced Materials* **2003,** *15* (22), 1881-1890.
15. Jothi, M.; Kumaradhas, P., Exploring the effect of metal electrodes and the transport properties of 4,4′-Di-prop-1-ynyl-biphenyl molecular nanowire using quantum chemical calculation and charge density study. *Computational and Theoretical Chemistry* **2012,** *995*, 79-91.
16. Dolgov, L.; Pidhirnyi, D.; Dovbeshko, G.; Lebedieva, T.; Kiisk, V.; Heinsalu, S.; Lange, S.; Jaaniso, R.; Sildos, I., Graphene-Enhanced Raman Scattering from the Adenine Molecules. *Nanoscale Research Letters* **2016,** *11* (1), 197.
17. Neese, F.; Wennmohs, F.; Becker, U.; Riplinger, C., The ORCA quantum chemistry program package. *The Journal of Chemical Physics* **2020,** *152* (22).
18. Talbi, D.; Chandler, G. S., Theoretical infrared spectra of biphenyl, terphenyls and tetraphenyls for astrophysical purposes. *Journal of Molecular Spectroscopy* **2012,** *275*, 21-27.

19. Gebre, S. T.; Martinez-Gomez, L.; Miller, C. J.; Kubiak, C. P.; Ribeiro, R. F.; Lian, T., Fano Resonance in CO2 Reduction Catalyst Functionalized Quantum Dots. *Journal of the American Chemical Society* **2025,** *147* (13), 10966-10973.
20. https://webbook.nist.gov/cgi/cbook.cgi?ID=C7732185&Type=IR-SPEC&Index=1.
21. Hale, G. M.; Querry, M. R., Optical Constants of Water in the 200-nm to 200-μm Wavelength Region. *Appl. Opt.* **1973,** *12* (3), 555-563.
22. Venyaminov, S. Y.; Prendergast, F. G., Water (H2O and D2O) Molar Absorptivity in the 1000–4000 cm−1Range and Quantitative Infrared Spectroscopy of Aqueous Solutions. *Analytical Biochemistry* **1997,** *248* (2), 234-245.
23. Nowak, M. J.; Lapinski, L.; Kwiatkowski, J. S.; Leszczyński, J., Molecular Structure and Infrared Spectra of Adenine. Experimental Matrix Isolation and Density Functional Theory Study of Adenine 15N Isotopomers. *The Journal of Physical Chemistry* **1996,** *100* (9), 3527-3534.
